# Genomics analysis and degradation characteristics of lignin by *Streptomyces thermocarboxydus* strain DF3-3

**DOI:** 10.1186/s13068-022-02175-1

**Published:** 2022-07-12

**Authors:** Fangyun Tan, Jun Cheng, Yu Zhang, Xingfu Jiang, Yueqiu Liu

**Affiliations:** 1 School of Landscape Architecture, Beijing University of Agriculture, Beijing, 102206 People’s Republic of China; 2 School of Bioscience and Resource Environment, Beijing University of Agriculture, Beijing, 102206 People’s Republic of China; 3 Institute of Plant Protection, Chinese Academy of Agricultural Sciences, Beijing, 100193 People’s Republic of China

**Keywords:** *Streptomyces thermocarboxydus* strain DF3-3, Alkali lignin, Enzyme activity, Genomics, Metabolic pathways

## Abstract

**Background:**

Lignocellulose is an important raw material for biomass-to-energy conversion, and it exhibits a complex but inefficient degradation mechanism. Microbial degradation is promising due to its environmental adaptability and biochemical versatility, but the pathways used by microbes for lignin degradation have not been fully studied. Degradation intermediates and complex metabolic pathways require more study.

**Results:**

A novel actinomycete DF3-3, with the potential for lignin degradation, was screened and isolated. After morphological and molecular identification, DF3-3 was determined to be *Streptomyces thermocarboxydus*. The degradation of alkali lignin reached 31% within 15 days. Manganese peroxidase and laccase demonstrated their greatest activity levels, 1821.66 UL^−1^ and 1265.58 UL^−1^, respectively, on the sixth day. The highest lignin peroxidase activity was 480.33 UL^−1^ on the fourth day. A total of 19 lignin degradation intermediates were identified by gas chromatography–mass spectrometry (GC–MS), including 9 aromatic compounds. Genome sequencing and annotation identified 107 lignin-degrading enzyme-coding genes containing three core enzymatic systems for lignin depolymerization: laccases, peroxidases and manganese peroxidase. In total, 7 lignin metabolic pathways were predicted.

**Conclusions:**

*Streptomyces thermocarboxydus* strain DF3-3 has good lignin degradation ability. Degradation products and genomics analyses of DF3-3 show that it has a relatively complete lignin degradation pathway, including the β-ketoadipate pathway and peripheral reactions, gentisate pathway, anthranilate pathway, homogentisic pathway, and catabolic pathway for resorcinol. Two other pathways, the phenylacetate–CoA pathway and the 2,3-dihydroxyphenylpropionic acid pathway, are predicted based on genome data alone. This study provides the basis for future characterization of potential biotransformation enzyme systems for biomass energy conversion.

**Supplementary Information:**

The online version contains supplementary material available at 10.1186/s13068-022-02175-1.

## Background

Lignocellulosic biomass is an easily available, low cost and renewable feedstock for biofuel which is an important direction for the development of renewable energy. Currently, the process for pretreatment of lignocellulosic biofuel requires removal or delocalization of lignin, which might generate aromatic compounds that inhibit enzymatic hydrolysis and fermentation [1]. Due to its rich aromatic content, lignin is a valuable waste from the biomass industry [2]. Researchers worldwide are focusing on lignin and its components for conversion into value-added products. Lignin is a complex aromatic heteropolymer derived from the condensation of hydroxyphenylpropane monomers and comprises various ether bonds and carbon–carbon bonds [3]. It is mainly composed of three basic monomers: guaiacyl (G) units, syringyl (S) units and p-hydroxyphenyl (H) units [4, 5]. Microorganisms that degrade lignocellulose are widely distributed in nature, and known examples include bacteria and fungi. Among them, fungi and some bacteria have mainly been studied, and white-rot fungi, and brown rot fungi in particular have obvious degradation effects on lignin [6, 7], but the development of industrial applications has been difficult [8]. Some bacteria also participate in lignin degradation. Several typical lignin-degrading bacteria, such as *Rhodococcus*, *Pseudomonas, Sphingobium* and *Sphingomonas,* have been identified [9–12]. *Streptomyces* are among the identified bacteria capable of degrading lignin [13]. Pasti et al. [14] isolated 11 strains of actinomycetes from the intestines of termites and analysed their ability to degrade lignocellulose, lignin and carbohydrates. Other researchers have also screened streptomyces from the soil that degrade lignin [15–17].

There are many kinds of enzymes involved in the degradation of lignin, including laccase (Lac), lignin peroxidase (LiP), manganese peroxidase (MnP), multifunctional peroxidase (VP), and dye decolouring peroxidase (DyPs) [18]. The specific mechanism for biological degradation of lignin needs to be studied further. Lignin is rich in high-value degradation intermediates, such as vanillin, guaiacol, catechin and protocatechin [19]. At the same time, the study of the metabolic mechanism of lignin and its transformation and utilization is an important part of the research needed for production of “second-generation biofuels” [20].

Hundreds of lignin derivatives have been identified in studies of bacterial degradation of lignin [21]. Because of the complex degradation mechanism, the interpretation of metabolic pathways and intermediate products is an important part of understanding the degradation of lignin. With the development of sequencing technology and bioinformatics, genomics research has become an important method for studying degradation mechanisms. Researchers are paying increasing attention to analysing metabolism of intermediate products through annotations of related degradation genes. *Pseudomonas putida* is a lignin-degrading bacterium that has been studied earlier. Lin et al. [22] identified several lignin-degrading enzymes, including haem peroxidase, from its genome and constructed five lignin metabolic pathways. Masai et al. [23] conducted a series of studies on lignin degradation and related genes in *Sphingomonas paucimobilis* SYK-6 and established a relatively complete lignin degradation metabolic pathway. Niewerth et al. [24] determined and analysed the whole genome sequence of *Arthrobacter* sp. Rue61a in soil, and the results showed that it has an aromatic degradation pathway that utilizes the characteristic products of lignin degradation, reflecting the saprophytic capabilities and nutritional diversity of organisms.

Several Streptomyces strains, such as *Streptomyces viridosporus* T7A [25] and *Streptomyces setonii* 75Vi2 [26], have been reported to degrade lignin. However, the actual catabolic pathways of lignin derivatives and the responsible enzymes and genes have not been investigated using molecular methods with *Streptomyces*. In our present study, a novel isolate, DF3-3, identified as *Streptomyces thermocarboxydus*, was found to degrade lignin. Alkali lignin is a model compound with a structure similar to that of lignocellulose and is often used as a raw material for lignin degradation studies [27]. This study used alkali lignin to investigate the characteristics of alkali lignin degradation by *Streptomyces thermocarboxydus* strain DF3-3. GC–MS combined with genomics was used to identify the genes responsible for lignin degradation and explore the metabolic pathway for lignin degradation by *Streptomyces thermocarboxydus* strain DF3-3.

## Results

### Morphological and physiological characteristics of DF3-3

**Fig. 1:**
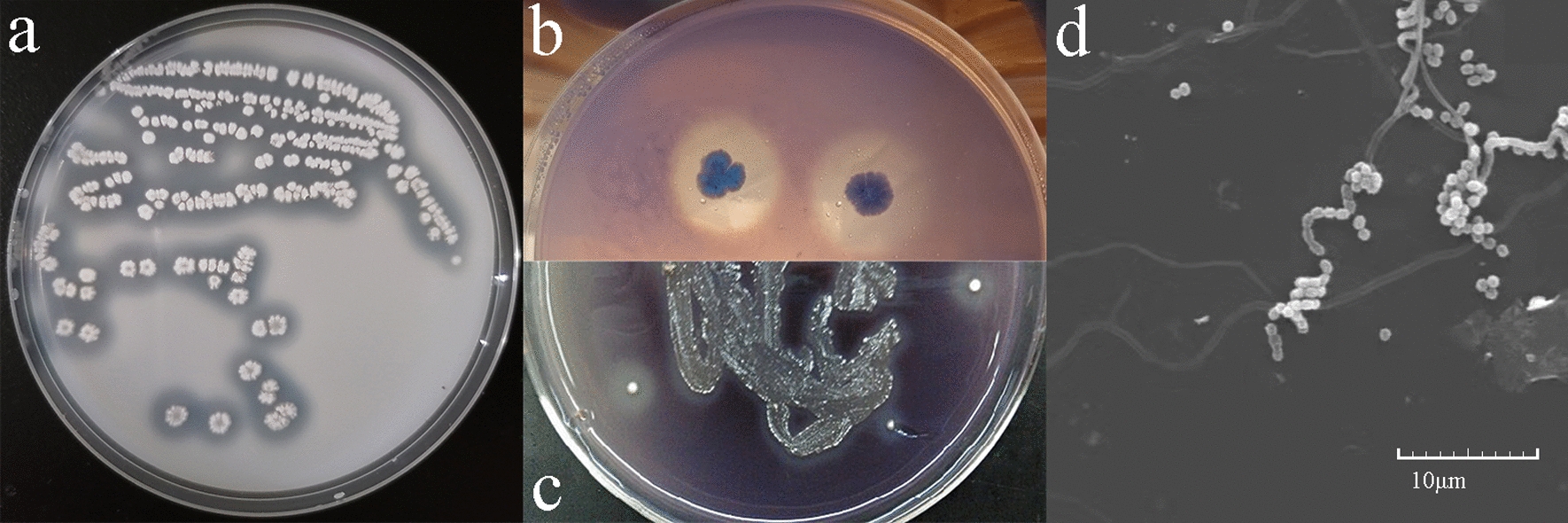
DF3-3 colour reaction and morphological characteristics. **a** Colony morphology streaked on a plate of DF3-3. **b** Transparent fading circle on Gause’s–Azure B medium. **c** Colour reaction on Gause’s–guaiacol medium**. d** Scanning electron micrograph of DF3-3

A strain of actinomycete named DF3-3 was isolated from the greening litter of Beijing University of Agriculture. The scribing form of the plate shows a rough white surface, the upper and lower surfaces are inconsistent in colour, and the hyphae in the base are obvious on Gause's medium (Fig. 1a). When DF3-3 was inoculated on Gause’s guaiacol medium, a clear colour reaction appeared. When inoculated on Gause’s–Azure B medium, a transparent fading circle appeared (Fig. 1b, c). This shows that DF3-3 has the ability to degrade lignin [28]. Microscopy was used to observe the morphology of DF3-3 (Fig. 1d). DF3-3 grew luxuriantly on the plate, hyphae were developed, and aerial hyphae were slender. The spore filaments were spiral-shaped with obvious characteristics of *Streptomyces*. Figure 1d shows a scanning electron micrograph of DF3-3 grown on Gause's medium. Mature spore chains were moderately long, with 40 to 80 spores per chain. The single spores were oval or cylindrical with diameters of 0.5 to 0.7 μm and lengths of 1.1 to 1.3 μm and have rough surfaces.

While keeping the conditions of the basal medium otherwise unchanged, different nitrogen sources and carbon sources were added to observe the growth of DF3-3 with different nitrogen sources. The results are shown in Table 1. DF3-3 could grow on six common nitrogen sources including ammonium chloride, potassium nitrate, ammonium sulfate, ammonium tartrate, acrylamide and peptone, and could grow on sixteen common carbon sources including glucose, mannose, melibiose, starch, maltose, α-D-methyl glucoside, trehalose, cellobiose, xylose, salicin, glycerol, sodium malate, sodium succinate, sodium malonate, sodium tyrosinate,Amylase. However, it could not grow on other eleven common carbon sources (Table 1). DF3-3 was inoculated onto the Gause’s liquid medium, and incubated, respectively, at different temperatures and pH conditions, the optimum temperature range of 30–35 °C and optimum pH 7.5–8.5 were found by examining the strain weights after 7 days (Additional file 1: Fig. S2).

**Table 1:** Utilization characteristics of the nitrogen source and carbon source of DF3-3

Trait	Result	Trait	Result	Trait	Result
Nitrogen source					
NH_4_Cl	+	(NH_4_)_2_SO_4_	+	Acrylamide	+
Potassium nitrate	+	Ammonium tartrate	+	Peptone	+
Carbon source					
Glucose	+	Alpha-D-methyl glucoside	+	Sodium lactate	−
Mannose	+	trehalose	+	Sodium acetate	−
Melibiose	+	Cellobiose	+	Sodium formate	−
L-arabinose	−	Xylose	+	Sodium malate	+
Starch	+	Ribose	−	Sodium succinate	+
Melezitose	−	Inulin	−	Sodium malonate	+
Erythritol	−	Salicin	+	Sodium tartrate	−
Maltose	+	Glycerin	+	Sodium tyrosine	+
Sucrose	−	Sodium butyrate	−	Amylase	+

+ Growth on carbon sources and nitrogen sources, respectively,—no growth

### Molecular identification

Genome de novo sequencing was performed on DF3-3 cells using second- and third-generation sequencing methods, namely, Illumina HiSeq + PacBio, and the gene location and sequence information of the samples were obtained through de novo assembly and gene prediction. According to the whole genome sequencing results (Table 2), strain DF3-3 has a chromosome with a total genome length of 7,311,713 bp. GeneMarkS predicted and annotated a total of 6929 coding sequences (CDSs), with a G + C content of 72.24%.

**Table 2:** Genome-wide characteristics of DF3-3

	value
Genome size (bp)	7,311,713
Chrom No	1
GC content (%)	72.24
Gene No	6929
GC content in gene region (%)	72.52
Gene average Len(bp)	927.29
Gene/genome (%)	87.88
GC content in intergenetic region (%)	70.17
tRNA No	66
rRNA No	18

**Fig. 2:**
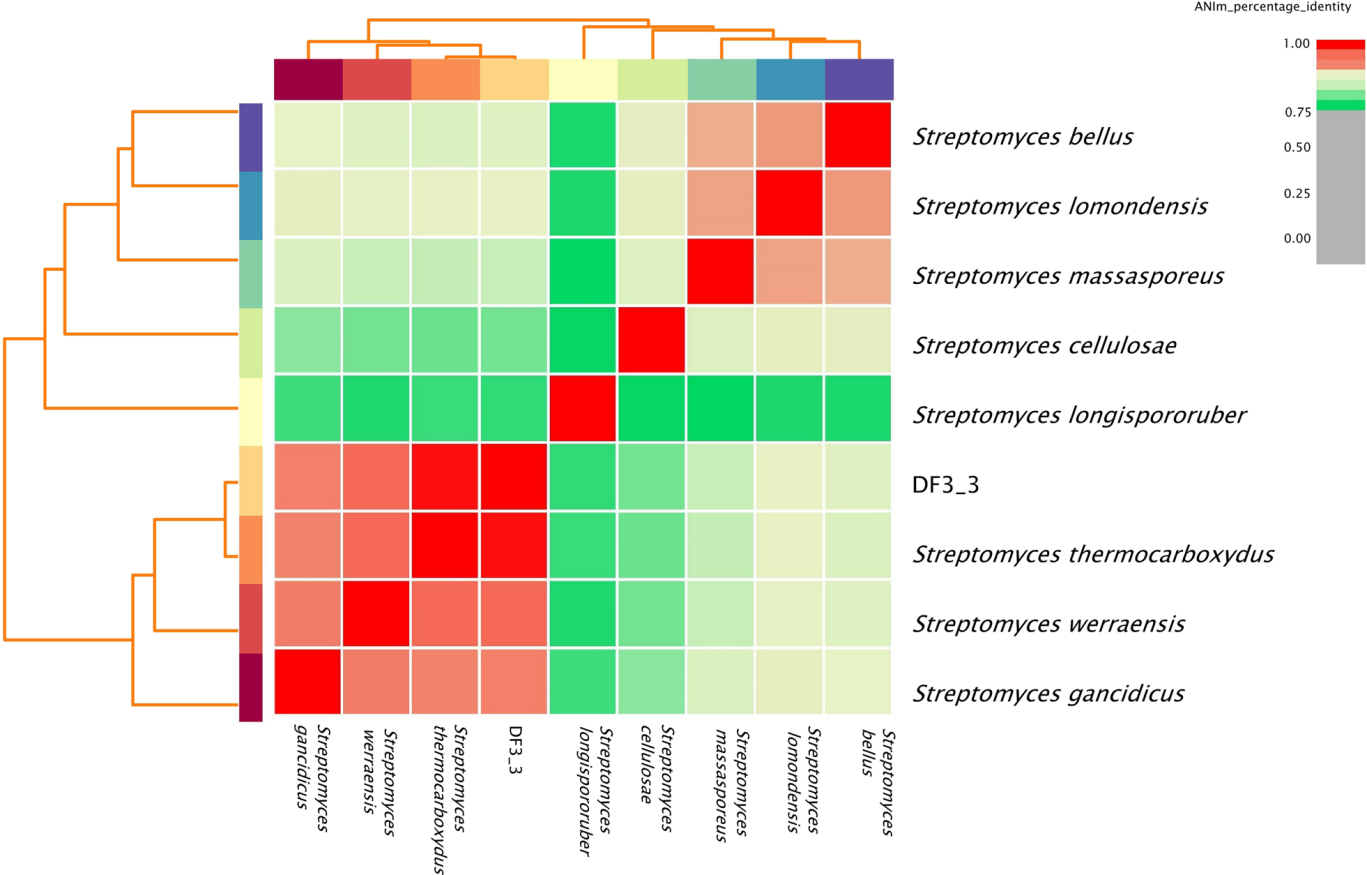
ANI analysis results for DF3-3 and other bacteria

At present, 95% of the average nucleotide identity (ANI) is often used as the standard for species classification and species clustering [29]. The whole genomes of eight strains with high homology to strain DF3-3 were selected and compared and analysed with DF3-3 using ANI. The results are shown in Fig. 2. The similarity between DF3-3 and *Streptomyces thermocarboxydus* reached 98.96%, and it can now be identified as *Streptomyces thermocarboxydus*.

### Biodegradation of alkali lignin by DF3-3

**Fig. 3:**
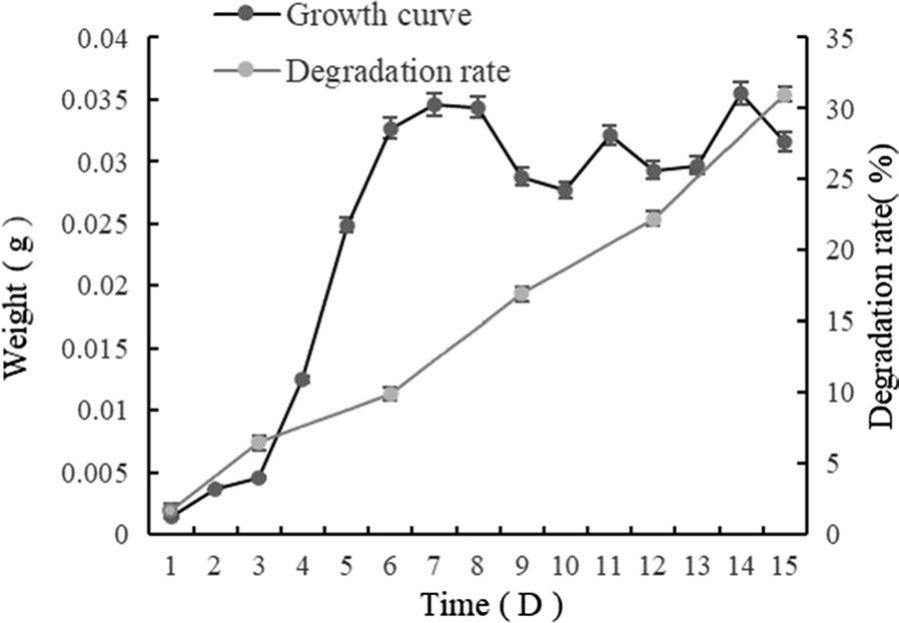
Growth curve and alkaline lignin degradation curve for DF3-3. Average values of three replicates are shown with the standard error of the mean as error bars

To investigate lignin degradation by strain DF3-3, cells were incubated at 30 °C in medium with alkali lignin as the carbon source. The growth curve and degradation rate of alkali lignin are shown in Fig. 3. The growth rate of DF3-3 increased significantly in 1–7 days, then, maintained a small fluctuation in 8–14 days. The degradation rate of alkaline lignin maintained a relatively uniform increase in days 1–14, eventually, the degradation rate reached a maximum value of 31% on day 15. The degradation of lignin by microorganisms requires a relatively slow process to reach a significant level. Fungi are more efficient in the breakdown of lignin than bacteria in which delignification is slower and more limited [30], the white-rot fungus *Phanerochaete chrysosporium* was used for degradation of lignin, and the efficiency reached approximately 20% on day 15 [31], so DF3-3 exhibited a better performance. This result is also similar to the degradation results seen with some *Streptomyces* strains, such as *S. viridosporus* T7A (lignin loss 30.9%) and *S. setonii* 75Vi2 (lignin loss 34.1%) [26, 32].

### Analysis of lignin-degrading enzymes

**Fig. 4:**
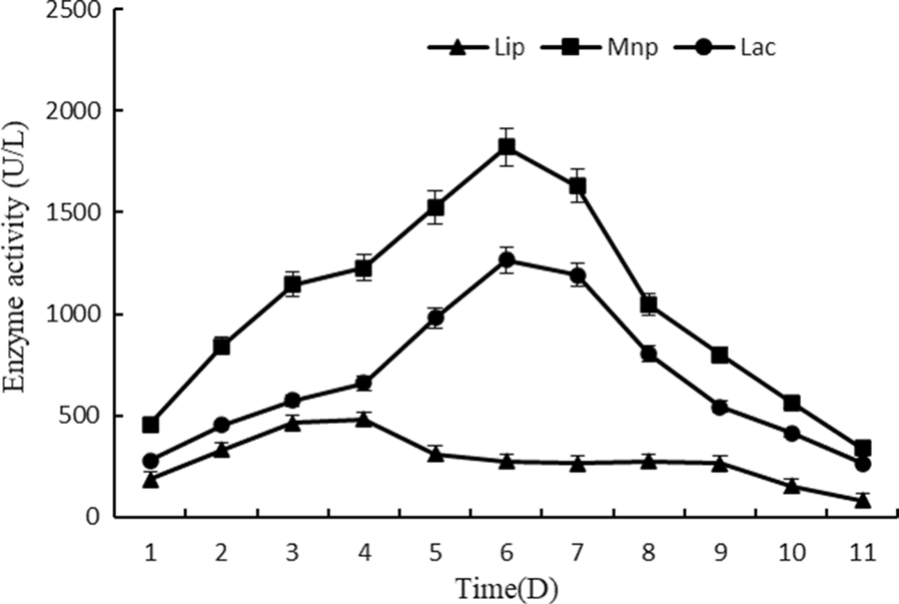
Enzyme activities of LiP, Lac, and MnP of DF3-3 during 11 days of incubation. Average values of three replicates are shown with the standard errors of the mean shown as error bars

Lignin molecules are not easily taken passively into the cell; therefore, *Streptomyces thermocarboxydus* DF3-3 might produce extracellular enzymes for synergistic degradation. Three major types of enzymes responsible for the degradation of lignin are lignin peroxidase (LiP), manganese peroxidase (MnP) and laccase (Lac) [33]. MnPs oxidize Mn(II) to Mn(III), and Mn(III) oxidizes phenolic compounds and generates phenoxy radicals that in turn undergo a variety of reactions, resulting in depolymerization. In the presence of Mn(II), MnP oxidizes nonphenolic lignin model compounds via peroxidation of unsaturated lipids. LiP is the most effective peroxidase and can oxidize phenolic and nonphenolic compounds, amines, aromatic ethers, and polycyclic aromatics [34]. Lac is a copper oxidoreductase [35] that can degrade refractory polyphenols and nonphenolics in lignin, and the expression of its coding genes in bacteria has also been reported [36]. The activities of these three enzymes from DF3-3 are shown in Fig. 4. MnP and Lac activity increased constantly during the initial 6 days, with maxima of 1821.66 U/L and 1265.58 U/L seen at day 6, followed by slight decreases from day 7. Lip activity was maintained at a low level, with a maximum of 480.33 U/L on day 4. These results indicated that MnP and Lac play crucial roles during the entire process of alkaline lignin degradation by DF3-3.

### Aromatic intermediates identified by GC–MS analysis

Using ethyl acetate as the solvent for GC–MS determination [37], the degradation products of alkali lignin produced in incubation with *Streptomyces thermocarboxydus* strain DF3-3 were analysed from the first day to the 15th day. In total, 19 degradation intermediate products were identified by GC–MS, including seven different types of compounds, including eight organic acids (butanoic acid (**4**), lactic acid (**8**), 2-hydroxypropanoic acid (**9**), pyrrole-2-carboxylic acid (**10**), 3-phenylpyruvic acid (**12**), 4-hydroxybenzoic acid (**13**), 4-hydroxyphenylpyruvate (**14**), palmitic acid (**16**), stearic acid (**17**)), three esters (acetic acid butyl ester (**1**), dibutyl phthalate (**15**), bis(2-ethylhexyl) phthalate (**19**)), two ethers (1-(1-ethoxy)propane (**2**) and 1-(1-propoxyethoxy)propane (**6**)), two alcohols (2-ethoxyethanol (**5**) and ethylene glycol (**7**)), three aromatic hydrocarbons (m-xylene (**3**)), 2,4-Di-tert-butylphenol (**11**) and 2,2′-Methylenebis(6-tert-butyl-4-methyl-phenol) (**18**). These 19 low molecular weight products are related to lignin metabolism, and their serial numbers, name, and retention times are shown in Table 3.

**Table 3:** Compounds identified from degradation of alkali lignin by DF3-3

	Retention time	Compounds	Molecular formula	Control group
1	4.530	Acetic acid, butyl ester	C_6_H_12_O_2_	+
2	4.651	1-(1-Ethoxyethoxy) propane	C_7_H_16_O_2_	+
3	5.690	M-Xylene	C_8_H_10_	+
4	6.055	Butyric acid	C_4_H_8_O_2_	+
5	6.251	2-Ethoxyethanol	C_4_H_10_O_2_	+
6	6.642	1-(1-Propoxyethoxy) propane	C_8_H_18_O_2_	+
7	8.793	Ethylene glycol	C_2_H_6_O_2_	+
8	11.058	Lactic acid	C_3_H_6_O_3_	+
9	13.038	2-Hydroxybutyric acid	C_4_H_8_O_3_	−
10	19.375	Pyrrole-2-carboxylic acid	C_5_H_5_NO_2_	−
11	23.225	2,4-Di-tert-butylphenol	C_14_H_22_O	−
12	25.121	3-Phenylpyruvic acid	C_9_H_8_O_3_	−
13	25.996	4-Hydroxybenzoic acid	C_7_H_6_O_3_	−
14	32.028	4-Hydroxyphenylpyruvate	C_9_H_8_O_4_	−
15	32.970	Dibutyl phthalate	C_16_H_22_O_4_	+
16	34.543	Palmitic acid	C_16_H_32_O_2_	+
17	38.084	Stearic acid	C_18_H_36_O_2_	+
18	41.008	2,2'-Methylenebis(6-tert-butyl-4-methyl-phenol)	C_23_H_32_O_2_	−
19	43.033	Bis(2-ethylhexyl) phthalate	C_24_H_38_O_4_	−

“ + ” indicates that the product was detected in the uninoculated control group, “−” indicates that the product was not detected in the uninoculated control group

The process of lignin degradation can be divided into two main parts. First, through the rupture of aromatic ether bonds and carbon–carbon bonds, lignin polymers are depolymerized to form various oligomers. Then, these depolymerized aromatic organic compounds form other small molecular benzene compounds [38]. Here, 9 aromatic compounds, including m-xylene (**3**), pyrrole-2-carboxylic acid (**10**), 2,4-di-tert-butylphenol (**11**), 3-phenylpyruvic acid (**12**), 4-hydroxybenzoic acid (**13**), 4-hydroxyphenylpyruvate (**14**), dibutyl phthalate (**15**), 2,2′methylenebis(4-methyl-6-tert-butyl phenol) (**18**), and bis(2-ethylhexyl) phthalate (**19**) were detected among the products produced by DF3-3 in degradation of alkaline lignin. Dibutyl phthalate (**15**) is a common lignin degradation intermediate [39–41] that can be metabolized to produce phthalic acid, which is further converted into syringyl, protocatechin and other phenolic compounds and then degraded [42]. According to the metabolic pathway for formation of dibutyl phthalate (**15**) and diisooctyl phthalate (**19**), product **19** is thought to have a similar degradation process.

Both 4-hydroxyphenylpyruvate (**14**) and 3-phenylpyruvic acid (**12**) were detected among the products of the experimental group, and their aromatic structures were related to alkali lignin. 4-Hydroxybenzoic acid is an important intermediate in many lignin metabolic pathways, including the coumaric acid pathway, gentisic acid pathway, and cinnamic acid pathway [43]. This indicated that DF3-3 uses the β-ketoadipate metabolic pathway or other similar degradation pathways.

According to the GC–MS results, 2,4-di-tert-butylphenol (**11**) and 2,2'-methyl bis(4-methyl-6-tert-butyl phenol) (**18**) were involved in the metabolic processes of DF3-3. Recent studies have also shown that there is a 2,4-di-tert-butylphenol metabolic pathway for microbial degradation of lignin [44]. Considering the resorcinol pathway and its correlative gene, it is speculated that there may be a similar pathway in DF3-3. It may retain the structure of the tert-butyl side face and be metabolized by the meta-cleavage pathway. The formation of 2,2'-methyl bis(4-methyl-6-tert-butyl phenol) (**18**) may come from the same metabolic intermediate as 2,4-di-tert-butylphenol. However, there are few studies on microbial degradation of this kind of structure at present. As an environmental pollutant, 4-tert-butylphenol can be degraded by several reported bacteria [45, 46]. However, to better understand this purification process, its specific metabolic process and some of the enzymes involved require further study.

### Analyses of the lignin metabolism pathways based on whole genome sequencing and annotation

#### Initial degradation of lignin into low molecular weight compounds by extracellular phenoloxidases

To identify the genes related to lignin degradation by DF3-3, the genes encoding known lignin enzymes of other bacterial species were selected as query sequences, and a BLASTp search of the DF3-3 genome was carried out. The results showed that DF3-3 has abundant genes encoding lignin-degrading enzymes, and a total of 107 different genes involved in the degradation process have been annotated (Table 4, Additional file 1: Tables S6–12).

**Table 4:** Main genes responsible for lignin degradation in DF3-3

Gene ID	Size (aa)	Gene name	Encode protein	Species of reference gene	Accession No. (NCBI)	BLAST identity (%)
gene 0557	599	ubiX	Aromatic acid decarboxylase	*Streptomyces* sp. 4F	ALV54514.1	97.99
gene 0904	1400	YhjG	Pentachlorophenol monooxygenase	*Streptomyces* sp. 4F	ALV51870.1	95.49
gene 1050	533	ahpD	Alkyl hydroperoxide reductase	*Actinospica acidiphila*	WP_163089316.1	99.44
gene 1234	1451	katE	Catalase	*Streptomyces* sp. UNC401CLCol	WP_028959332.1	99.79
gene 2609	989	–	Multicopper oxidase domain-containing protein	*Streptomyces* sp. XHT-2	WP_161108161.1	99.39
gene 2768	1091	–	Aryl-alcohol dehydrogenase	*Streptomyces* sp. di50b	SCD35921.1	93.66
gene 2849	962	pdxA	Terephthalate dihydrodiol dehydrogenase	*Streptomyces* sp. McG7	WP_215047915.1	98.75
gene 2857	983	–	Phenoxybenzoate dioxygenase	*Streptomyces tuirus*	WP_190903372.1	88.07
gene 2915	2288	copA	Carbonate dehydratase	*Actinospica acidiphila*	WP_058917537.1	96.19
gene 3045	383	–	Extradiol dioxygenase	*Streptomyces* sp. 4F	ALV53653.1	93.70
gene 3220	2276	katE	Catalase HPII	*Streptomyces* sp. XHT-2	WP_161108660.1	99.74
gene 3600	1502	–	2-Polyprenyl-6-methoxyphenol hydroxylase	*Streptomyces* sp. di50b	SCD47389.1	85.06
gene 3744	959	–	Pimeloyl-ACP methyl ester carboxylesterase	*Streptomyces* sp. di188	SCD58877.1	88.43
gene 3898	2225	katG	Catalase-peroxidase	*Streptomyces* sp. Akac8	WP_136237873.1	99.87
gene 3899	380	fur	Transcriptional repressor	*Streptomyces* sp. UNC401CLCol	WP_028958816.1	99.21
gene 3983	1655	katE	Catalase	*Streptomyces* sp. GESEQ-13	WP_210638384.1	99.09
gene 4491	197	–	Multicopper oxidase domain-containing protein	unclassified *Streptomyces*	WP_106959434.1	98.46
gene 5116	1529	aldH	Aldehyde dehydrogenase	*Streptomyces* sp. XHT-2	MXQ60577.1	99.61
gene 5348	716	yfiH	LACCASE	*Streptomyces griseorubens*	GGQ64023.1	96.64
gene 5542	1025	–	Trans-1,2-dihydrobenzene-1,2-diol dehydrogenase	*Streptomyces afghaniensis* 772	EPJ42281.1	82.27
gene 5998	2267	copA	Carbonate dehydratase	*Streptomyces* sp. UNC401CLCol	WP_028959238.1	99.74
gene 6181	1415	glcD	FAD-linked oxidase	*Actinospica acidiphila*	NEC50278.1	98.73
gene 6937	1277	–	Dye-decolorizing peroxidase	*Streptomyces pharetrae* CZA14	OSZ61955.1	83.61

In the initial stages of lignin degradation, extracellular enzymes are responsible for depolymerization of lignin. Lignin-modifying enzymes (LME) and lignin-degrading auxiliary (LDA) enzymes are two main groups of lignin-degrading enzymes [47]. In this study, multiple lignin depolymerization-related genes were found in the genome of DF3-3 (Table 4), including genes encoding LME, i.e., multicopper oxidase genes (gene 5348, gene 4491, gene 2609), dye-decolorizing peroxidase gene (gene 6937), manganese peroxidase-encoding genes (gene 3898, gene 3899), and genes encoding LDA, i.e., alcohol dehydrogenase genes (gene 2768, gene 0557, gene5543), alcohol dioxygenase gene (gene 3045), catalase genes (gene 1234, gene 3983, gene 3220), aldehyde dehydrogenase (gene 5116), pentachlorophenol monooxygenase (gene 0904) and terephthalate dihydrodiol dehydrogenase (gene 2849).

#### Catabolism pathways for lignin components

**Fig. 5:**
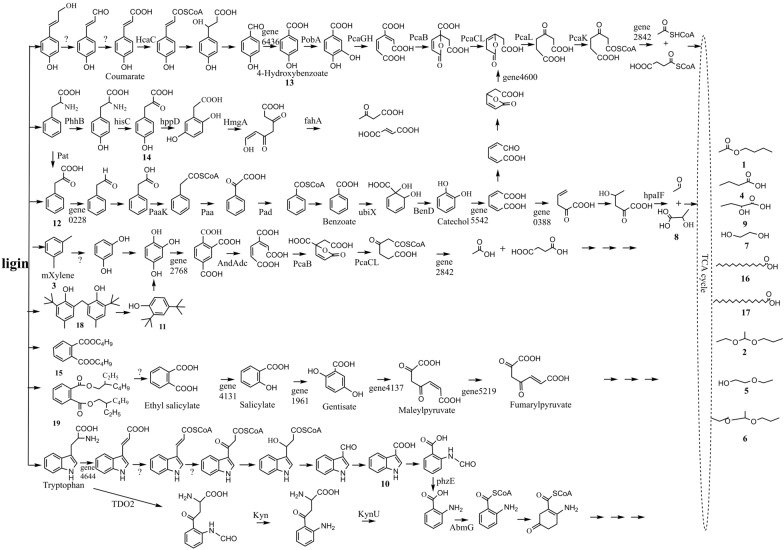
Predicted lignin degradation pathway for *Streptomyces thermocarboxydus* strain DF3-3. Numbers used to represent the compounds are the same as those in Table 3

**Fig. 6:**
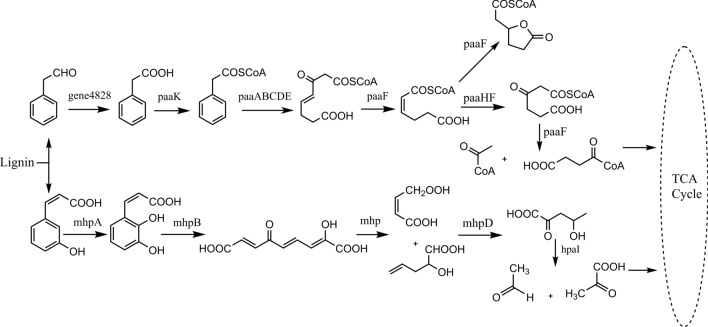
Lignin metabolic pathway of DF3-3 to be further verified

Many low molecular weight compounds are produced by initial depolymerization of lignin. Based on GC–MS and genome analysis, 5 metabolic pathways of lignin-based derivatives were predicted: β-ketoadipate pathway and peripheral reactions; gentisate pathway; anthranilate pathway; homogentisic pathway; and catabolic pathway for resorcinol (shown in Fig. 5). Based on genome data alone, we predicted two other pathways: the phenylacetate–CoA pathway and the 2,3-dihydroxyphenylpropionic acid pathway (Fig. 6).

##### β-Ketoadipate pathway and peripheral reactions

**Fig. 7:**
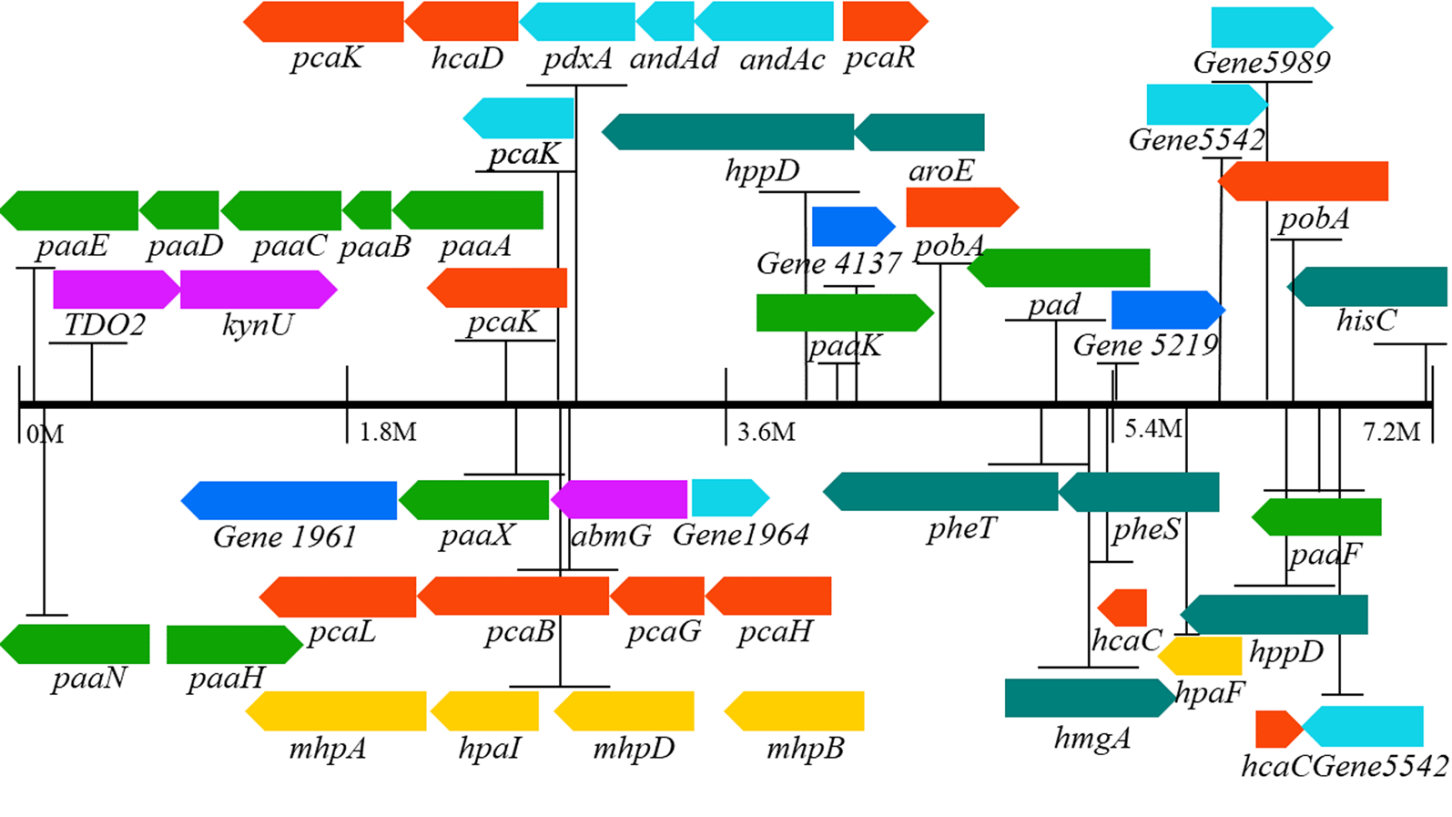
Locations of genes encoding aromatic catabolic pathways are indicated in the genome of DF3-3; orange, genes for the β-ketoadipate central pathway; green, genes for the phenylacetyl-CoA ring-cleavage pathway; yellow, genes for the 2,3-dihydroxyphenylpropionate pathway; purple, genes for the anthranilate pathway; dark green, genes for the homogentisate pathway; dark blue, genes for the gentisate pathway; blue, genes for the resorcinol pathway

The β-ketoadipate pathway is an important aromatic metabolic pathway for many microorganisms. There are 26 genes involved in the degradation process of lignin in DF3-3 (Additional file 1: Table S6), and they are scattered throughout the genome of DF3-3 (Fig. 7). The key genes of the protocatechuic acid 3,4-dioxygenase (pcaG, pcaH) gene appear in the whole genome of DF3-3 and can be imagined to metabolize lignin intermediates through the β-ketoadipate pathway branched by protocatechin. There are two 4-hydroxycinnamic acid dioxygenases (hcaC, hcaD) in DF3-3, and 4-hydroxycinnamic acid can initially be degraded to generate 4-hydroxycinnamic acid coenzyme A and further converted into p-hydroxybenzoic acid, which was found among the degradation products of alkaline lignin produced by DF3-3. 3-Phenylpyruvic acid and 4-hydroxyphenylpyruvate were also detected as metabolites of DF3-3. We speculate that they can also be degraded by the hcaC gene. Then, 4-hydroxyphenylpyruvate undergoes a series of reactions to produce p-hydroxybenzoic acid. The pcaG and pcaH genes cause benzene epoxidation to enable a ring-opening reaction generating cis-hexadienedioic acid; this further generates β-ketoadipate, which is degraded and finally enters the tricarboxylic acid cycle. Unlike 4-hydroxyphenylpyruvate, 3-phenylpyruvic acid may not be converted only into 4-hydroxyphenylpyruvate by hcaC but could also be converted into benzoic acid and catechol, and then a ring-opening reaction takes place to enter the β-ketoadipate pathway or generate 4-hydroxy-2-oxovalerate and further degradation.

##### The gentisate pathway

The gentisate pathway usually starts from the degradation of salicylic acid. Ethyl salicylate is a precursor in the production of salicylic acid. The pathway for degradation of phthalic acid by some fungi has been explained, in which dibutyl phthalate or diisooctyl phthalate is converted to ethyl salicylate [48]. Some aromatic oxygenases may play important roles in this process. In the DF3-3 genome, the genes encoding salicylic acid-5-hydroxylase (gene 1961) and gentisic acid 1,2-dioxygenase (gene 4137) were identified (Additional file 1: Table S7). Gentianic acid 1,2-dioxygenase oxygenates the benzene ring of gentisic acid to produce maleylpyruvate, which is then further metabolized by maleate pyruvate isomerase (gene 5219). Dibutyl phthalate and diisooctyl phthalate were detected among the metabolites of DF3-3. Phthalate dioxygenase oxygenase (gene 4131) detected in the DF3-3 genome gene also indicated that it may appear as an intermediate product of the salicylic acid metabolic pathway.

##### The anthranilate pathway

The specific mechanism of the anthranilic acid pathway in bacteria has not been fully elucidated [43, 49]. A 2-aminobenzoate-CoA ligase (abmG) and an L-kynurenine hydrolase (kynU) gene were identified in DF3-3, and a tryptophan 2,3-dioxygenase (TDO2) gene was also observed (Additional file 1: Table S8). It is speculated that the anthranilic acid pathway of DF3-3 generates formyl kynurenine through the degradation of tryptophan, which is further converted to kynurenine and then generates anthranilic acid under the action of kynU. In addition, tryptophan may undergo a series of redox reactions at its side chain to produce indole derivatives. The pyrrole-2-carboxylic acid detected among the metabolites of DF3-3 can also be degraded by the anthranilate synthase (phzE) gene and abmG gene through a similar pathway.

##### Catabolic pathway for resorcinol

Due to the existence of the metabolite m-xylene and related genes (Additional file 1: Table S9), it is speculated that DF3-3 has related metabolic pathways. The resorcinol pathways for other strains involve the participation of oxidoreductases. The functions of some of these genes, such as aromatic compound monooxygenase (gene 3600)-coding genes, have not been fully clarified. Aryl-alcohol dehydrogenase and extradiol dioxygenase may affect the degradation of aromatic phenols. It is speculated that the metabolism of m-xylene by DF3-3 may take place through oxidation of methyl groups to produce resorcinol. Then, resorcinol undergoes further oxygenation on the branch chain and ring-opening oxidation with the action of oxygenases (Fig. 5).

##### Homogentisic pathway

The homogentisic pathway is the central pathway for catabolism of phenylalanine and tyrosine. Genomic analysis of DF3-3 shows that phenylalanine is converted to tyrosine by phenylalanine hydroxylase and auxiliary methanolamine dehydratase (PhhB), and tyrosine aminotransferase (hisC) is converted to tyrosine 4-hydroxyphenylpyruvate, which is further converted into homogentisate by 4-hydroxyphenylpyruvate dioxygenase (hppD) and degraded by the action of homogentisate 1,2-dioxygenase (hmgA) to produce maleacetoacetate (Fig. 7). A fumarylacetoacetate hydrolase (fahA) gene was identified in the DF3-3 genome (Additional file 1: Table S10), indicating that hydrolysis of maleacetoacetate occurs through the degradation of fumarate acetoacetate. The genes involved in the homogentisate pathway are scattered throughout the genome, and the processes are carried out with the joint action of hmgA and other related genes. This non-linkage also appears in other bacteria [49]. 4-Hydroxyphenylpyruvate, as the precursor of homogentisic acid, was observed among the products detected by GC–MS, and two hppD genes observed in the genome are related to the 4-hydroxy phenylpyruvate dioxygenase in *Streptomyces cellulosae* and *Actinospica acidiphila* with high similarities (97.35% and 98.69%).

In addition to the above metabolic pathways, the following two possible lignin degradation pathways were found for DF3-3 through a gene search. A relatively complete set of coding genes is present in the genome to form the pathway, but the related metabolites were not observed in this study.

##### Phenylacetate–CoA pathway

Phenylacetate can be derived from lignin-related phenylpropane units [50], and the general pathway for aerobic metabolism has just been discovered and studied with some bacteria [51, 52]. There were at least 15 genes in DF3-3 involved in this process (Additional file 1: Table S11). The phenylacetate coenzyme A oxygenase gene is organized into clusters (paaABCDE) and exists in the DF3-3 genome (Fig. 7), downstream from which there are a phenylacetic acid degradation protein (paaN) and a 3-hydroxyacyl-CoA dehydrogenase (paaH) encoding gene; a 1,2-epoxyphenylacetyl-coenzyme A isomerase (paaG) gene exists a little further downstream, and the isomerase it encodes can convert 3-hydroxypropionyl-CoA into 1,2-epoxyphenylacetyl-CoA. At the same time, 3-hydroxypropionyl-CoA can also be further degraded by 3-hydroxypropionyl-CoA dehydrogenase (paaH) and 3-hydroxypropionyl-CoA dehydrogenase (paaF) and finally enter the tricarboxylic acid cycle.

##### 2,3-Dihydroxyphenylpropionic acid pathway

The 2,3-dihydroxyphenylpropionic acid pathway-related mhpABD gene cluster was observed in the DF3-3 genome (Additional file 1: Table S12), including encoding for 3-(3-hydroxy-phenyl) propionate hydroxylase (mhpA), 2,3-dihydroxyphenylpropionate 1,2-dioxygenase (mhpB) and fumarate acetoacetate hydrolase (mhpD) genes, which are located upstream of the 4-hydroxy-2-oxovalerate aldolase (hpaI) gene (Fig. 7). DF3-3 lacks the mhpE and mhpF genes, and the generated 4-hydroxy-2-oxovalerate is decomposed by the aldolase encoded by the hpaI gene. It was also determined that other aldehyde dehydrogenases may affect the degradation of acetaldehyde and generate pyruvate and acetyl-CoA to enter the tricarboxylic acid cycle for metabolism.

## Discussion

Biofuel production by biodegradable lignocellulose has far-reaching prospects [13]. Research on the degradation system of lignocellulose-degrading microorganisms has great significance to its practical application and development. However, few microorganisms, only white rot fungi, have been reported that can degrade lignin and cellulose at the same time [18]. In the actual degradation of biomass materials, the degradation of lignin, cellulose and hemicellulose often occurs simultaneously and is interrelated. In our research, we found that DF3-3 has good degradation performance on both lignin and cellulose. More experiments and new approaches are needed in future work to better develop and apply DF3-3.

The enzymology for bacterial lignin degradation has been well-studied in recent years, and some bacterial specific enzymes for lignin degradation have been reported. Like Cα-dehydrogenase (LigD) [53], glutathione-dependent β-etherase enzymes (LigE, F, G) have been identified from *Sphingomonas paucimobilis* SYK-6 [54], a demethylase enzyme (LigX) from *Pseudomonas paucimobilis* [55], and DyP-type peroxidases from *Rhodococcus jostii* RHA1 [56]. Recent reports have shown multicopper oxidases that demonstrate laccase activities [57, 58]. In our study, we found that DF3-3 had Lac, Mnp and Lip activities (Fig. 4). Genome research identified three multicopper oxidase coding genes (gene 5348, gene 4491, gene 2609), and gene 5348 has high similarity (96.64%) to the laccase structural protein gene of *Streptomyces griseorubens* (GGQ64023.1) [59], showing that DF3-3 has the ability to encode laccase at the genetic level. No gene encoding manganese peroxidase has been detected, but a catalase/peroxidase gene (gene3898) was found in DF3-3, which was 99.87% similar to KatG from *Streptomyces* sp. Akac8 [8]. The fur gene (gene3899) encoding a transcription regulator appears downstream, and these were reported as possible manganese peroxidase-encoding genes in *Streptomyces reticuli* [48, 49], suggesting that DF3-3 can exhibit manganese peroxidase activity. In addition, a gene (gene 6937) encoding a dye decolouring peroxidase was observed. The peroxidase encoded by it has a broad spectrum of substrates, which is also commonly reported in the depolymerization of lignin in some bacteria [22, 50, 51]. These peroxidases are even regarded as bacterial equivalents of fungal lignin peroxidase [47, 56]. *Rhodococcus jostii* RHA1, which produces DyP, was proved to be able to degrade lignin through β-ketoadipic acid pathway without hydrogen peroxide [60]. A lot of evidence of this degradation pathway was found in DF3-3.

The hydrogen peroxide-producing enzyme system mainly participates in lignin degradation as lignin-degrading auxiliary (LDA) [52]. They cannot degrade lignin directly, but they are essential to the whole degradation process [61]. These enzymes include glyoxal oxidase, aryl alcohol oxidase, quinone reductase and related dehydrogenases [53]. They produce hydrogen peroxide to support degradation by other peroxidases. The aryl-alcohol dehydrogenase (gene2768), and alcohol dioxygenase (gene 3045) annotated in the DF3-3 genome are thought to be involved in the process of lignin degradation. Especially aryl alcohol oxidase can produce hydrogen peroxide needed in lignin degradation by oxidative dehydrogenation of aryl alcohol, polyunsaturated primary alcohol or aromatic secondary alcohol [62]. In addition, catalase removes the hydrogen peroxide produced by these reactions quickly enough to prevent oxidative damage to [4Fe-4S]-clusters in proteins and protect the body from toxification [24, 54]. The catalase-encoding gene (katE) identified in DF3-3 is thought to be involved in lignin degradation.

The lignin degradation pathway of actinomycetes was first studied through research on the culture medium and metabolites [32, 63]. As the understanding of molecular biology increased, people began to seek more direct evidence. Masai et al. [23] first established a relatively complete pathway of lignin degradation and metabolism by means of enzymology and genomics. In *Sphingomonas paucimobilis* SYK-6, β-aryl ether cleavage catalysis [64], the biphenyl ring cleavage pathway [65], the ferulate catabolic pathway [66], the O-demethylation systems of vanillate and syringate [67], the protocatechuate 4,5-cleavage pathway [68], and multiple 3-*O*-methylagallate catabolic pathways [69] were described. Eleven lignin metabolic pathways were found in the genome of *Cupriavidus necator*, among which the β-ketoadipate pathway also included four branches: catechol, chlorocatechol, methylcatechol and protocatechuate ortho ring-cleavage [43]. In our study, evidence of a lignin degradation pathway was found in the metabolites and genetics. The products detected by GC–MS are also related to five lignin metabolic pathways. However, some unusual products detected may point to new branches. We speculate that 4-hydroxyphenylpyruvate (**14**) and 3-phenylpyruvic acid (**12**) may be transformed from phenylalanine and enter the homologous pathway and β-ketoadipate pathway, respectively, for further metabolism. A large number of genes related to the β-ketoadipate pathway have been detected in DF3-3, but there are still some genes involved in the reaction process that have not been compared, and for some genes, the reaction process in which they specifically participate has not been further elucidate. Perhaps there are related reactions in DF3-3 which are different from those in other bacteria. The heterologous expression of these gene fragments is helpful to the establishment and production of efficient engineered bacteria with biological enzymes.

Studies have shown that bacterial degradation of lignin has complex growth condition-specific regulation [70]. Because of the diversity of structure of lignan compounds, different reactions may occur in the degradation process of different substrate. Based on genome data alone, we predicted two other pathways the phenylacetate–CoA pathway and the 2,3-dihydroxyphenylpropionic acid pathway, but did not find the related metabolites by GC–MS. This absence might be related to the substrates. To further verify the degradation process, transcriptome analysis, proteomic analysis and other biological methods are needed to study the enzymes and the genes involved in their degradation pathways to understand the biological function of DF3-3 in the degradation of lignin.

Interestingly, recent reports have demonstrated that some low-molecular-weight compounds occur during lignin degradation may play important roles in lignin degradation. For example, tryptophan residues and phenylalanine residues appeared to be the most important in the long range electron transfer process of lignin degradation [71]. Organic acids, phenoxy free radicals and various transition metal coordination complexes can act as diffusion media of electrons and react directly with lignin, causing bond breakage [72]. A large number of similar low molecular weight compounds were detected in the lignin degradation products of DF3-3, which may be involved in both degradation agents and products during the degradation process. Low molecular weight compounds, containing phenolic hydroxyl, methoxy, amine, ketone, aldehyde, carboxyl and other functional groups, can generate phenoxy radicals to oxidize the non-phenolic residues of lignin in the presence of redox enzymes [73]. The roles of these compounds in the degradation of lignin by DF3-3 need further investigation.

## Conclusions

Based on the above data and analyses, we isolated a bacterial strain identified as *Streptomyces thermocarboxydus* strain DF3-3 from greening litter and concluded that it degraded alkaline lignin, and the degradation efficiency reached 31% within 15 days. In total, 19 alkaline lignin degradation intermediates were identified by GC–MS, and 107 possible lignin-degrading enzyme encoding genes in the DF3-3 genome were annotated, 7 pathways for metabolism of lignin and its intermediates were predicted, including the β-ketoadipate pathway and peripheral reactions, gentisate pathway, anthranilate pathway, catabolic pathway for resorcinol, homogentisic pathway, phenylacetate–CoA pathway, and the 2,3-dihydroxyphenylpropionic acid pathway. Intermediates in the first five metabolic pathway were detected by GC–MS. The degradation products and genomics analyses show that DF3-3 has a relatively complete lignin degradation pathway.

## Methods

### Sampling, isolation and screening of bacterial strain

Samples were collected from greening litter of Beijing University of Agriculture (40.0947°N, 116.3151°E). One gram of sample was placed in a 50-mL sterile centrifuge tube containing 10 mL of sterile distilled water and shaken at 120 rpm overnight. Next, 10^−1^ and 10^−2^ serial dilutions of each sample suspension were spread as 0.1-mL aliquots on Gause’s synthetic medium with the formula (g/L): 0.5 NaCl; 1 KNO_3_; 0.5 K_2_HPO_4_·3H_2_O; 0.5 MgSO_4_·7H_2_O; 0.01 FeSO_4_·7H_2_O; 20 soluble starch [8]. The plates were incubated at 30 °C for 1 week, and distinct colonies were picked and subcultured for further analysis. Gause’s guaiacol medium and Gause’s–Azure B medium used for lignin degradation screening contained 0.1% guaiacol and 0.1% aniline blue, respectively, added to Gause’s medium. Different external nitrogen sources (20 g/L), such as acrylamide and potassium nitrate, and additional carbon sources (1 g/L), such as glucose and mannose, were used to replace soluble starch culture strains in studies of their utilization of nitrogen sources and carbon sources. The culture medium for detecting lignin degradation and enzyme activity was kraft lignin–MSM medium (3 g of kraft lignin L, 2 g of [NH_4_]_2_SO_4_, 1 g of K_2_HPO_4_, 1 g of KH_2_PO_4_, 0.2 g of MgSO_4_, 0.1 g of CaCl_2_, 0.05 g of FeSO_4_, and 0.02 g of MnSO_4_ in 1 L distilled water, pH 7.0).

### Scanning electron microscope observations

The shapes of the bacteria were observed by scanning electron microscopy. A cover glass was inserted into the solid medium to cultivate the strain, and the insert was removed after the bacterial body climbed onto the glass slide. The precipitate was washed by adding a phosphate buffer solution (pH 7.2), added to 2.5% glutaraldehyde, fixed at room temperature for 2–4 h, and then placed in a refrigerator at 4 °C overnight. After elution with a 30–95% ethanol gradient, the material was rinsed with tert-butanol, then 20 μL of tert-butanol was added and the mixture was put into a refrigerator at − 20 ℃ until it froze and solidified. Using critical point drying (HITACHI HCP-2 Critical Point Dryer) and gold sputter coating (Eiko IB-3 ion plating machine), the sample was observed by scanning electron microscopy (SEM, JSM-6360LV, JEOL, Japan) [74].

### Strain growth curve determination

To assess the growth of bacteria, an equal quantity of bacteria was inserted into Gause’s liquid medium and cultured on a shaker. The culture solution was removed and centrifuged every 24 h. The supernatant was discarded, and the filter paper was placed into an oven. The mixture was dried to a constant weight, and the filter paper and the bacteria were weighed. The weight of the filter paper was compared with the weight of the bacteria. All assays were performed with three replicates.

### Biodegradation of alkali lignin

To determine the lignin loss from alkaline lignin cause by various strains, samples (1.5 mL) were centrifuged at 12,000 ×*g* for 10 min. One millilitre of supernatant was diluted by adding 2 mL of phosphate buffer (pH 7.6). The lignin concentration was determined by measuring the absorbance at 280 nm with a UV–Vis spectrophotometer (Shimadzu UN-1900i) [75]. The calculated standard curve for lignin was y = 0.0786x–0.0245, *R*^2^ = 0.9988.

### Enzyme assay

Samples were centrifuged at 12,000 rpm for 5 min, and the supernatant was used for lignin peroxidase (Lip), laccase and manganese peroxidase (MnP) enzyme assays. Laccase activity was determined by monitoring the oxidation of ABTS at 420 nm (ε420 = 36,000 M^−1^ cm^−1^). A lignin peroxidase assay was carried out using peroxidase oxidation of Azure B. LiP activity was determined by measuring the absorbance at 651 nm (ε651 = 48.8 M^−1^ cm^−1^). Manganese peroxidase activity was determined from the change in absorbance occurring when Mn^2+^ is oxidized to Mn^3+^ and forms a complex with malonate, which produced absorbance at 270 nm (ε270 = 11,590 M^−1^ cm^−1^) [76].

### Genome sequencing and functional annotation

The genome of DF3-3 was sequenced at Major Biomedical Technology Co., Ltd. (Shanghai, China). Genomic DNA was extracted using a Wizard® Genomic DNA Purification Kit (Promega). Purified genomic DNA was quantified by a TBS-380 fluorometer (Turner BioSystems Inc., Sunnyvale, CA). The genome was sequenced by adopting the second-generation + third-generation sequencing method of Illumina HiSeq + PacBio, with a shotgun library of 400 bp insertion size. Assembly software canu, SPAdes, etc. was used for three-generation sequence assembly [77], and GeneMarkS software was used to predict the coding sequence (CDS) in the genome [78]. The prediction and annotation of genes were carried out using Prodigal Son (prokaryotic dynamic programming gene discovery algorithm). GeneMarkS was used to predict the plasmid genome. tRNAscan-SE v2.0 software was used to predict the tRNA contained in the genome, and Barrnap software was used to predict the rRNA contained in the genome. Functional annotation of the predicted coding gene was carried out by comparison with 6 major databases (NR, Swiss-Prot, Pfam, EggNOG, GO and KEGG) [79–82].

### Alkali lignin degradation products determined by GC–MS

DF3-3 was inoculated in 100 ml of medium with AL as the carbon source and cultured on a shaker for 7 days. Samples were collected every 24 h, and a number of control groups was set up. The sample was centrifuged (10,000 rpm, 15 min) to remove the bacteria, the supernatant was acidified with HCl to pH 2–3, and it was thoroughly extracted with a threefold volume of ethyl acetate. The extract was rotary evaporated to 10 ml at 37 °C and dried with anhydrous Na_2_SO_4_. After evaporating the solvent in a nitrogen stream, 100 µl of the organic layer was derivatized. Then, 100 μl of dioxane and 10 μl of pyridine were added to the sample and vortexed, and 50 μl of bis(trimethylsilyl)trifluoroacetamide (BSTFA) was added. The mixed solution was placed in a water bath at 80 °C for 45 min and shaken regularly. The silanized sample was tested by GC–MS [83].

The analytical column was a DB-5 capillary column (30 m length, 0.25 mm inner diameter, 0.25 mm film thickness). The carrier gas was helium. The column temperature was initially 50 °C (5 min), then it was raised to 280 °C (10 °C/min, holding time of 5 min). The transmission line and ion source temperatures were 200 and 250 °C, respectively. The solvent delay time was 4.0 min. The injection volume was 1 μl. Electron ionization mass spectra were recorded in the range 30–550 (m/z) in full scan mode.

## Supplementary Information

**Additional file 1: Figure S1. **Schematic of the *Streptomyces thermocarboxydus* strain DF3-3 genome. The first and fourth circles of the circle diagram from the outside to the inside are the CDS on the positive chain and the negative chain, and different colors indicate different COG functional classifications. The second and third circles are CDS, tRNA and rRNA; on positive chain and negative chain, respectively. The fifth circle is GC content. The outward part indicates that the GC content in this region is higher than the average GC content of the whole genome. The higher the peak value, the greater the difference from the average GC content. The inward part indicates that the GC content in this region is lower than the average GC content of the whole genome. The sixth circle is GC-Skew value, and the specific algorithm is G-C/G+C, which can assist in judging leading strand and lag chain. In general, leading strand GC skew>0 and lag chain GC skew < 0 can also assist in judging replication origin (minimum cumulative offset) and end point (maximum cumulative offset), especially for circular genome. The innermost circle is the genome size marker. **Figure S2. **Growth of DF3-3 at different culture temperature and pH. **a** Growth at different temperatures. **b** Growth at different pH values. **Table S1. **The weight (g) of the growth curve and the degradation rate of the alkaline lignin of DF3-3. **Table S2. **Enzyme activities (U/L) of LiP, Lac, and MnP of DF3-3. **Table S3.** GCMS spectrum of products of alkaline lignin degradation by DF3-3. **Table S4.** Compounds at each peak and their relative contents. **Table S5.** GCMS spectra of products of alkaline lignin degradation by DF3-3. **Table S6. **Genes responsible for the β-ketoadipate pathway and peripheral reactions. **Table S7. **Genes responsible for gentisate pathways. **Table S8. **Genes responsible for pathways for anthranilate. **Table S9. **Genes responsible for pathways for resorcinol. **Table S10.** Genes responsible for catabolic pathways for homogentisic. **Table S11. **Genes responsible for pathways for phenylacetate–CoA. **Table S12. **Genes responsible for catabolic pathways for 2,3-dihydroxyphenylpropionate.

## Data Availability

The additional data generated during this study are available in the Additional file***.***

## Abbreviations

AL: Alkali lignin
GC–MS: Gas chromatography–mass spectrometry
MnP: Manganese peroxidase
Lac: Laccase
LiP: Lignin peroxidase
