# Supplementary material for: Genomics analysis and degradation characteristics of lignin by Streptomyces thermocarboxydus strain DF3-3
Source: Biotechnol Biofuels Bioprod. 2022 Jul 12;15:78. doi: 10.1186/s13068-022-02175-1 (PMC9277890; doi:10.1186/s13068-022-02175-1)
Supplement: Supplementary file 1 — Additional file 1: Figure S1. Schematic of the Streptomyces thermocarboxydus strain DF3-3 genome. The first and fourth circles of the circle diagram from the outside to the inside are the CDS on the positive chain and the negative chain, and different colors indicate different COG functional classifications. The second and third circles are CDS, tRNA and rRNA; on positive chain and negative chain, respectively. The fifth circle is GC content. The outward part indicates that the GC content in this region is higher than the average GC content of the whole genome. The higher the peak value, the greater the difference from the average GC content. The inward part indicates that the GC content in this region is lower than the average GC content of the whole genome. The sixth circle is GC-Skew value, and the specific algorithm is G-C/G+C, which can assist in judging leading strand and lag chain. In general, leading strand GC skew>0 and lag chain GC skew < 0 can also assist in judging replication origin (minimum cumulative offset) and end point (maximum cumulative offset), especially for circular genome. The innermost circle is the genome size marker. Figure S2. Growth of DF3-3 at different culture temperature and pH. a Growth at different temperatures. b Growth at different pH values. Table S1. The weight (g) of the growth curve and the degradation rate of the alkaline lignin of DF3-3. Table S2. Enzyme activities (U/L) of LiP, Lac, and MnP of DF3-3. Table S3. GCMS spectrum of products of alkaline lignin degradation by DF3-3. Table S4. Compounds at each peak and their relative contents. Table S5. GCMS spectra of products of alkaline lignin degradation by DF3-3. Table S6. Genes responsible for the β-ketoadipate pathway and peripheral reactions. Table S7. Genes responsible for gentisate pathways. Table S8. Genes responsible for pathways for anthranilate. Table S9. Genes responsible for pathways for resorcinol. Table S10. Genes responsible for catabolic pathways for homogentis [file 13068_2022_2175_MOESM1_ESM.docx]

**Additional file 1**

**Genomics Analysis and Degradation Characteristics of Lignin by Streptomyces thermocarboxydus strain DF3-3**

**Author names and affiliations:**

Fangyun Tan^1^ ,Jun Cheng^2^, Yu Zhang^1^, Xingfu Jiang^3^ Yueqiu Liu^1＊^

*1 School of Landscape Architecture, Beijing University of Agriculture, Beijing 102206 , China*

*2 School of Bioscience and Resource Environment, Beijing University of Agriculture, Beijing 102206 , China*

*3 Institute of Plant Protection, Chinese Academy of Agricultural Sciences*, *Beijing* 100193, *China*

***Corresponding Author:** Yueqiu Liu

School of Landscape Architecture, Beijing University of Agriculture, Beijing, China

Tel: 86 010 80797210

Email: [liuyueqiu@bua.edu.cn](mailto:liuyueqiu@bua.edu.cn)

**First author:** Fangyun Tan

Email: [tanfangyun123@foxmail.com](mailto:tanfangyun123@foxmail.com)

Jun Cheng

Email:chengjun@bua.edu.cn

**Co-authors:** Yu Zhang

Email:1759250779@qq.com

Xingfu Jiang

xfjiang@ippcaas.cn


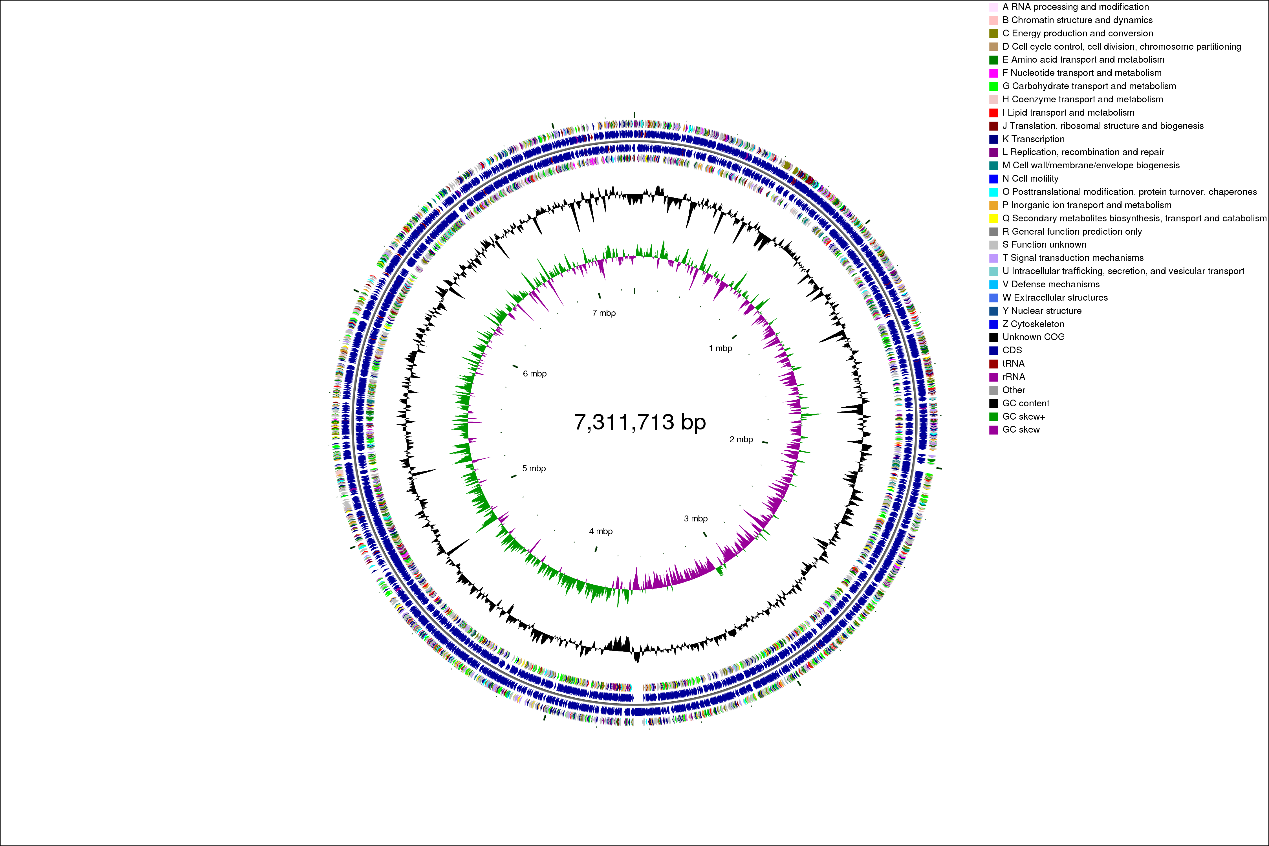


**Figure S1** Schematic of the *Streptomyces thermocarboxydus* strain DF3-3 genome.

The first and fourth circles of the circle diagram from the outside to the inside are the CDS on the positive chain and the negative chain, and different colors indicate different COG functional classifications. The second and third circles are CDS, tRNA and rRNA; on positive chain and negative chain respectively. The fifth circle is GC content. The outward part indicates that the GC content in this region is higher than the average GC content of the whole genome. The higher the peak value, the greater the difference from the average GC content. The inward part indicates that the GC content in this region is lower than the average GC content of the whole genome. The sixth circle is GC-Skew value, and the specific algorithm is G-C/G+C, which can assist in judging leading strand and lag chain. Generally, leading strand GC skew>0 and lag chain GC skew<0 can also assist in judging replication origin (minimum cumulative offset) and end point (maximum cumulative offset), especially for circular genome. The innermost circle is the genome size marker.

**
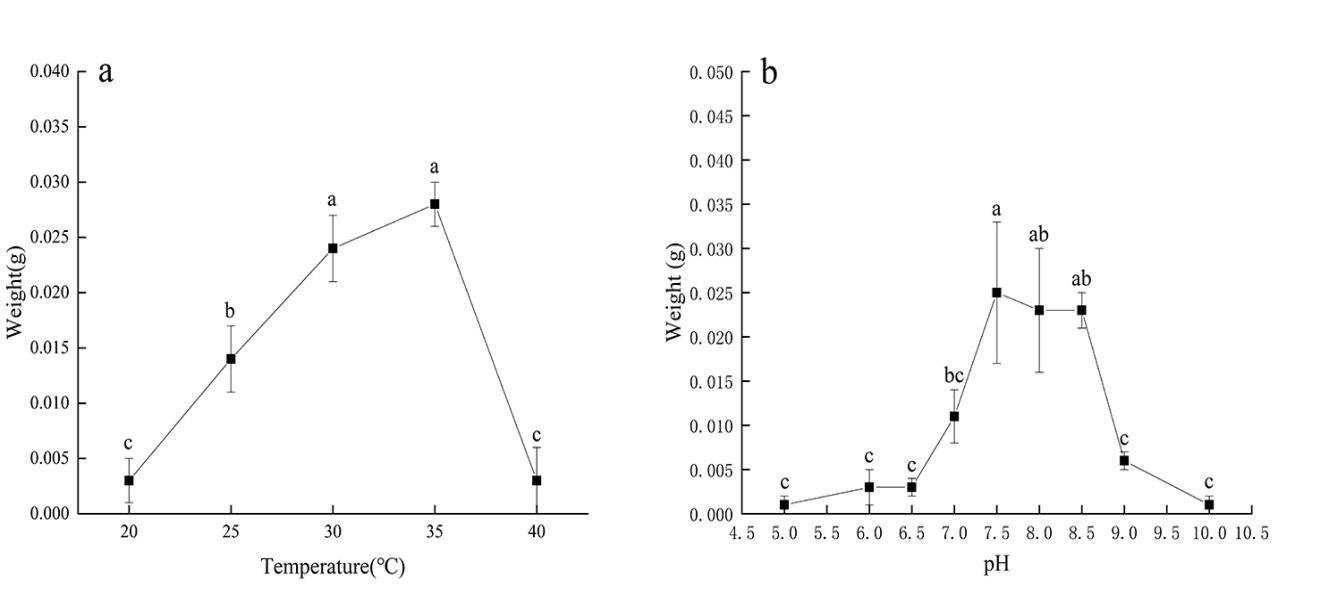
**

**Figure S2** Growth of DF3-3 at different culture temperature and pH.

**a** Growth at different temperatures. **b** Growth at different pH values.

**Table S1** The weight (g) of the growth curve and the degradation rate of the alkaline lignin of DF3-3.

| Item/Time (D) | Weight (g) | Degradation rate (%) |
| --- | --- | --- |
| 1 | 0.0015±0.001 | 1.67±1.33 |
| 2 | 0.0037±0.001 | - |
| 3 | 0.0046±0.003 | 6.44±2.52 |
| 4 | 0.0125±0.003 | - |
| 5 | 0.0249±0.008 | - |
| 6 | 0.0327±0.010 | 9.92±2.26 |
| 7 | 0.0346±0.002 | - |
| 8 | 0.0344±0.001 | - |
| 9 | 0.0288±0.006 | 16.91±0.45 |
| 10 | 0.0277±0.006 | - |
| 11 | 0.0321±0.009 | - |
| 12 | 0.0293±0.009 | 22.22±2.62 |
| 13 | 0.0297±0.004 | - |
| 14 | 0.0355±0.008 | - |
| 15 | 0.0316±0.008 | 31.01±3.62 |

**Table S2** Enzyme activities (U/L) of LiP, Lac, and MnP of DF3-3.

| Enzyme(U/L)/  Time(D) | Lip | Mnp | Lac |
| --- | --- | --- | --- |
| 1 | 184.50±12.91 | 454.32±22.71 | 276.39±13.81 |
| 2 | 332.12±23.24 | 842.16±42.10 | 452.84±22.64 |
| 3 | 462.27±32.35 | 1145.67±57.28 | 575.17±28.75 |
| 4 | 480.33±33.62 | 1226.71±61.33 | 658.34±32.91 |
| 5 | 311.46±21.80 | 1524.97±76.24 | 979.72±48.98 |
| 6 | 274.91±19.24 | 1821.66±91.08 | 1265.58±63.27 |
| 7 | 265.04±18.55 | 1631.24±81.56 | 1192.31±59.61 |
| 8 | 273.68±19.15 | 1045.28±52.26 | 803.47±40.17 |
| 9 | 264.55±18.51 | 797.44±39.87 | 542.26±27.11 |
| 10 | 153.21±10.72 | 562.11±28.10 | 411.82±20.59 |
| 11 | 411.82±20.59 | 339.72±16.98 | 264.11±13.20 |

**Table S**3 GCMS spectrum of products of alkaline lignin degradation by DF3-3.

| Time (D) | GCMS spectrum |
| --- | --- |
| 0 |  |
| 1 | **** |
| 2 | **** |
| 3 | **** |
| 4 | **** |
| 5 | **** |
| 6 | **** |
| 7 | **** |
| 8 | **** |
| 14 | **** |

**Table S4** Compounds at each peak and their relative contents.

| Peak No. | Compounds name | Molecular formula | Relative content (%) | | | | | | | | | |
| --- | --- | --- | --- | --- | --- | --- | --- | --- | --- | --- | --- | --- |
|  |  |  | 0 | 1 | 2 | 3 | 4 | 5 | 6 | 7 | 8 | 14 |
|  | Acetic acid, butyl ester | C_6_H_12_O_2_ | 3.81 | 3.32 | 2.34 | 1.86 | 2.55 | 2.97 | 3.51 | 3.68 | 2.59 | 2.87 |
|  | 1-(1-Ethoxyethoxy) propane | C_7_H_16_O_2_ | 20.12 | 16.87 | 12.88 | 12.41 | 11.01 | 12.78 | 15.74 | 15.51 | 11.76 | 14.35 |
|  | M-Xylene | C_8_H_10_ | 5.85 | 4.96 | 3.79 | 3.62 | 3.12 | 3.62 | 4.54 | 4.34 | 3.38 | 3.85 |
|  | Butyric Acid | C_4_H_8_O_2_* | 2.10 | 1.47 | 2.95 | 1.83 | 2.49 | 2.29 | 3.08 | 2.84 | 2.47 | 1.79 |
|  | 2-Ethoxyethanol | C_4_H_10_O_2_* | 6.56 | 4.51 | 5.28 | 3.97 | 3.77 | 3.74 | 5.45 | 5.67 | 4.38 | 4.33 |
|  | 1-(1-Propoxyethoxy) propane | C_8_H_18_O_2_ | 2.23 | 1.70 | 1.72 | 1.38 | 1.36 | 1.48 | 1.70 | 1.79 | 1.25 | 1.52 |
|  | Ethylene glycol | C_2_H_6_O_2_* | 32.76 | 18.95 | 29.67 | 20.18 | 19.57 | 19.89 | 29.39 | 32.69 | 23.79 | 24.84 |
|  | Lactic Acid | C_3_H_6_O_3_* | 1.31 | 3.02 | 1.15 | 2.32 | 0.89 | 1.55 | 0.60 | 0.47 | 0.52 | 0.17 |
|  | 2-Hydroxybutyric acid | C_4_H_8_O_3_* | 0 | 2.69 | 0.80 | 3.44 | 0.42 | 1.58 | 0.31 | 0.55 | 0.38 | 0.05 |
|  | Pyrrole-2-carboxylic acid | C_5_H_5_NO_2_ | 0 | 0.09 | 0.01 | 0.03 | 0.01 | 0.17 | 2.19 | 6.30 | 0.27 | 0.46 |
|  | 2,6-Di-t-butylphenol | C_14_H_22_O | 0 | 0.94 | 1.08 | 0.51 | 0.59 | 0.75 | 0.83 | 0.87 | 0.90 | 2.07 |
|  | 3-Phenylpyruvic acid | C_9_H_8_O_3_* | 0 | 0.22 | 2.68 | 16.54 | 19.53 | 3.98 | 0.72 | 0.02 | 16.97 | 4.72 |
|  | 4-Hydroxybenzoic acid | C_7_H_6_O_3_* | 0 | 0.40 | 0.56 | 1.56 | 0.99 | 1.36 | 0.55 | 0.44 | 0.66 | 1.57 |
|  | 4-Hydroxyphenylpyruvate | C_9_H_8_O_4_* | 0 | 0.34 | 0.54 | 0.97 | 1.60 | 0.42 | 0.48 | 0.58 | 4.31 | 3.02 |
|  | Dibutyl phthalate | C_16_H_22_O_4_ | 10.85 | 13.26 | 13.41 | 10.08 | 10.08 | 11.94 | 14.71 | 9.18 | 11.19 | 14.68 |
|  | Palmitic Acid | C_16_H_32_O_2_* | 8.12 | 6.12 | 7.11 | 12.54 | 7.18 | 8.81 | 9.70 | 9.94 | 9.43 | 14.22 |
|  | Stearic acid | C_18_H_36_O_2_* | 3.14 | 2.54 | 3.09 | 5.94 | 3.97 | 5.09 | 5.26 | 4.60 | 4.73 | 4.09 |
|  | 2,2'-Methylenebis(6-tert-butyl-4-methyl-phenol) | C_23_H_32_O_2_ | 0 | 17.98 | 10.50 | 0.39 | 10.55 | 16.24 | 0.83 | 0.28 | 0.64 | 0.74 |
|  | Bis(2-ethylhexyl) phthalate | C_24_H_38_O_4_ | 0 | 3.32 | 0.44 | 0.44 | 0.31 | 1.34 | 0.41 | 0.25 | 0.37 | 0.67 |

**Table S5** GCMS spectra of products of alkaline lignin degradation by DF3-3.

| No. | MS/MS pattern |
| --- | --- |
|  | 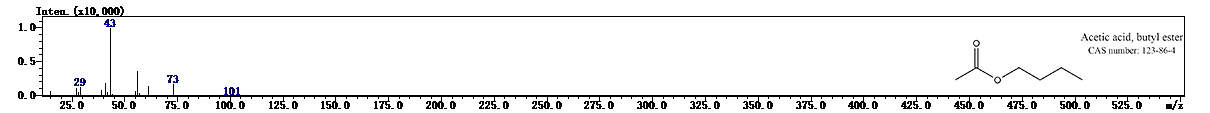 |
|  | 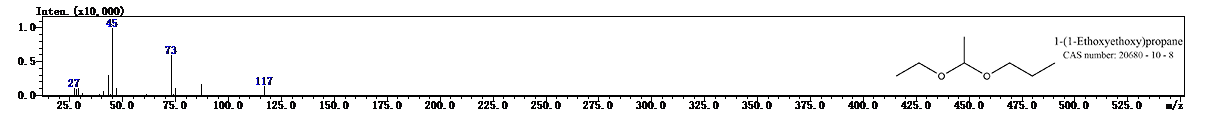 |
|  | 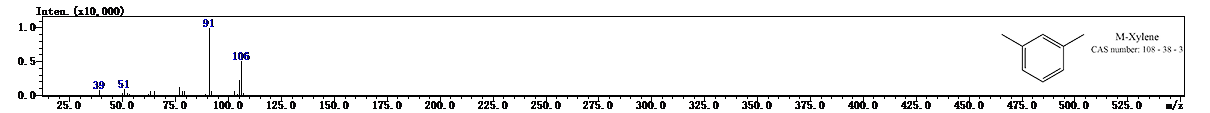 |
|  | 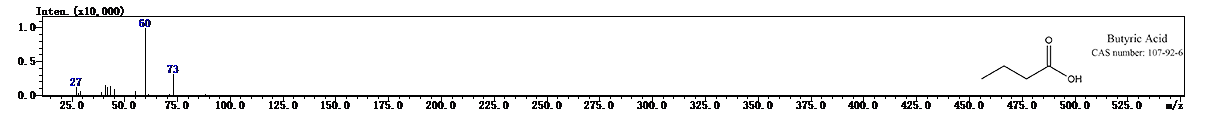 |
|  | 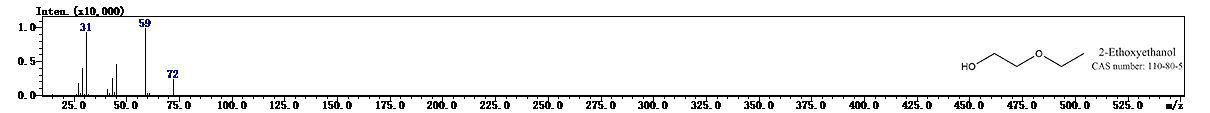 |
|  | 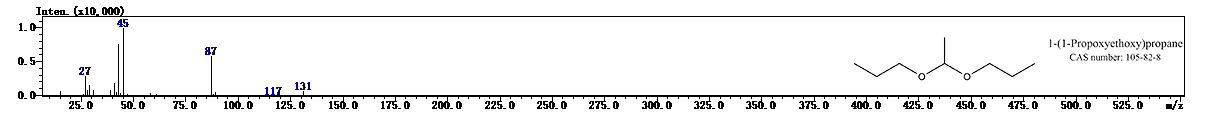 |
|  | 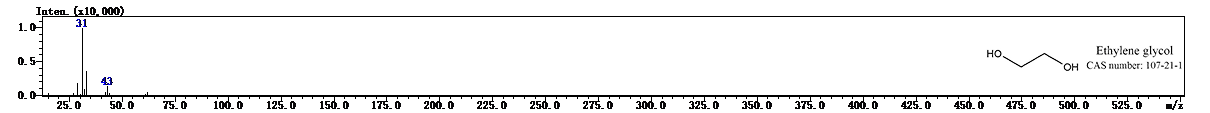 |
|  | 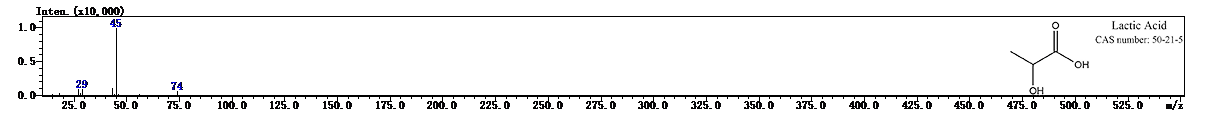 |
|  | 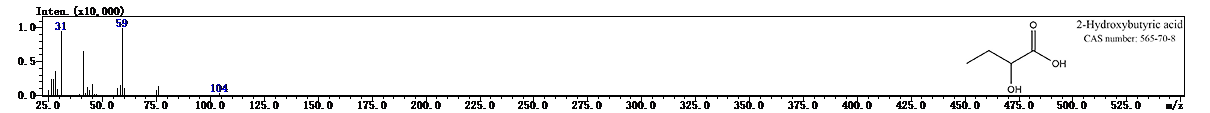 |
|  | 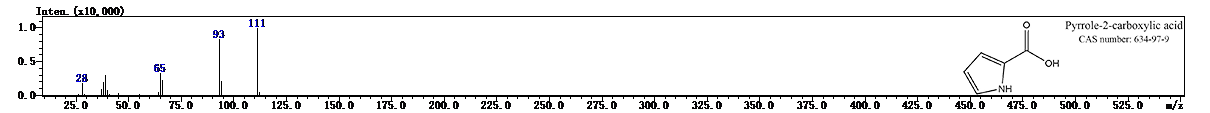 |
|  | 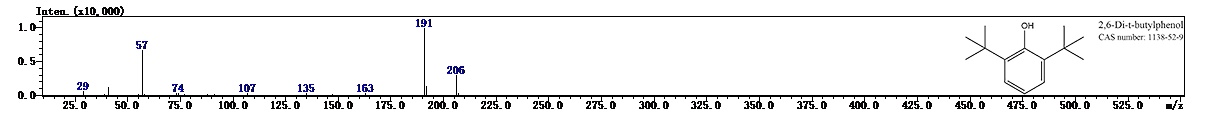 |
|  | 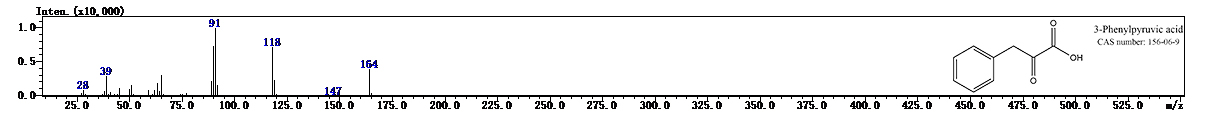 |
|  | 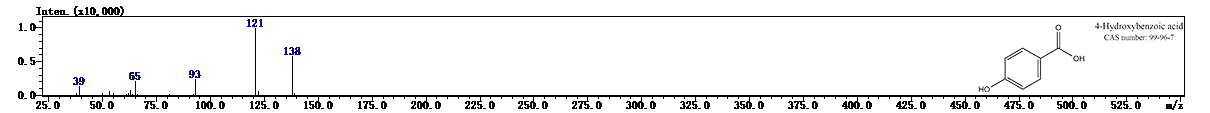 |
|  | 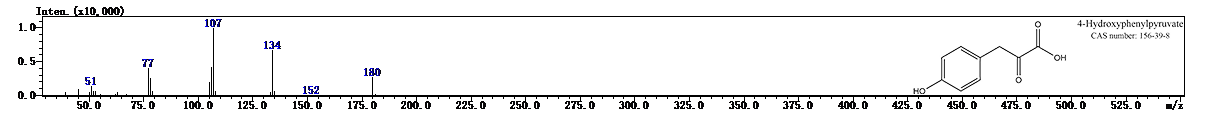 |
|  | 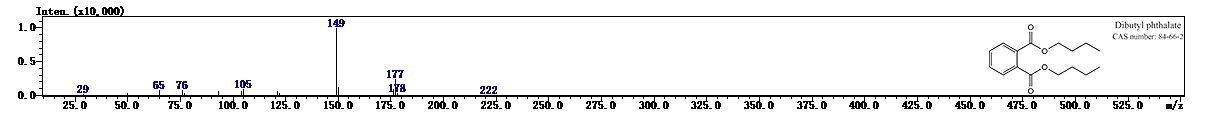 |
|  | 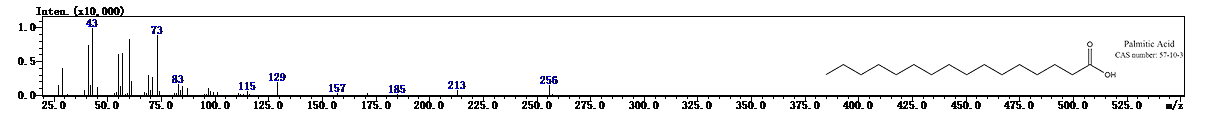 |
|  | 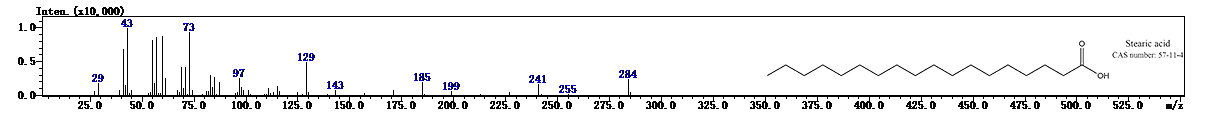 |
|  | 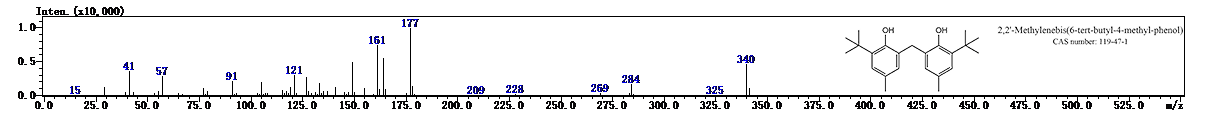 |
|  | 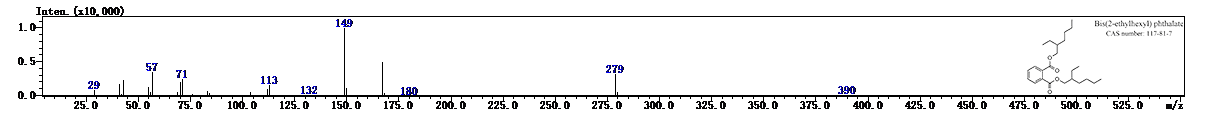 |

**Table S6** Genes responsible for the β-ketoadipate pathway and peripheral reactions.

| Gene ID | Size (aa) | Gene Name | Encode protein | Species of reference gene | Accession no. (NCBI) | BLAST identity (%) |
| --- | --- | --- | --- | --- | --- | --- |
| gene 3935 | 1001 | - | Vanillate O-demethylase oxygenase subuni | *Streptomyces* sp. SID4956 | WP_161232727.1 | 99.40 |
| gene 2826 | 773 | hpaI | 4-hydroxy-2-oxovalerate aldolase | *Streptomyces* sp. XHT-2 | MXQ61669.1 | 99.61 |
| gene 0228 | 335 | - | phenylpropionate dioxygenase-like ring-hydroxylating dioxygenase | *Streptomyces paradoxus* | MBB6077390.1 | 94.59 |
| gene 0557 | 599 | ubiX | aromatic acid decarboxylase | *Streptomyces* sp. 4F | ALV54514.1 | 97.99 |
| gene 0021 | 377 | pcaC | 4-carboxymuconolactone decarboxylase | *Streptomyces* sp. ok210 | WP_093930409.1 | 98.40 |
| gene 0859 | 506 | - | carboxymuconolactone decarboxylase family protein | *Streptomyces* sp. GESEQ-13 | WP_210634556.1 | 99.40 |
| gene 2323 | 1199 | pcaL | 4-carboxymuconolactone decarboxylase | *Streptomyces* sp. Akac8 | WP_136239018.1 | 99.75 |
| gene 2838 | 1121 | pcaL | 3-oxoadipate enol-lactonase | *Streptomyces* sp. SID4956 | WP_161232455.1 | 99.73 |
| gene 2839 | 1331 | pcaB | 3-carboxy-cis, cis-muconate cycloisomerase | *Streptomyces* sp. UNC401CLCol | WP_028959090.1 | 99.55 |
| gene 2840 | 605 | pcaG | protocatechuate 3,4-dioxygenase subunit alpha | *Streptomyces* sp. GESEQ-13 | WP_210638048.1 | 99.50 |
| gene 2841 | 773 | pcaH | protocatechuate 3,4-dioxygenase subunit beta | *Streptomyces* sp. GESEQ-13 | WP_210638047.1 | 99.22 |
| gene 2842 | 1208 | - | β-ketoadipyl CoA thiolase | *Streptomyces* sp. B9173 | OQR62711.1 | 88.25 |
| gene 2831 | 1364 | pcaK | 4-hydroxybenzoate transporter PcaK | *Streptomyces* sp. GESEQ-13 | WP_210638055.1 | 98.02 |
| gene 2847 | 1439 | pcaK | 4-hydroxybenzoate transporter PcaK | *Streptomyces* sp. Akac8 | WP_136238348.1 | 99.79 |
| gene 2852 | 734 | pcaR | Pca regulon regulatory protein | *Streptomyces* *cellulosae* | GHE61509.1 | 97.13 |
| gene 2848 | 1013 | hcaD | Terephthalate 1,2-dioxygenase | *Streptomyces cellulosae* | GHE61489.1 | 97.63 |
| gene 4139 | 1148 | hcaD | hypothetical protein | *Streptomyces* sp. GESEQ-13 | WP_210637914.1 | 98.43 |
| gene 5175 | 317 | hcaC | 3-phenylpropionate/trans-cinnamate dioxygenase ferredoxin subunit | *Streptomyces* sp. di50b | SCD40618.1 | 97.14 |
| gene 5716 | 1265 | hcaD | pyridine nucleotide-disulfide oxidoreductase | *Streptomyces* sp. 4F | ALV50055.1 | 96.67 |
| gene 5542 | 1025 | - | Trans-1,2-dihydrobenzene-1,2-diol dehydrogenase | *Streptomyces afghaniensis* 772 | EPJ42281.1 | 82.27 |
| gene 4140 | 923 | - | 4,5-dihydroxyphthalate decarboxylase | *Streptomyces* sp. 3212.3 | REE57871.1 | 84.69 |
| gene 4458 | 1220 | pobA | 4-hydroxybenzoate 3-monooxygenase | *Streptomyces* sp. XHT-2 | WP_161108189.1 | 99.01 |
| gene 6344 | 1175 | pobA | 4-hydroxybenzoate 3-monooxygenase | *Streptomyces* sp. XHT-2 | WP_161107724.1 | 99.49 |
| gene 6436 | 1517 | - | cyclohexanone monooxygenase | *Streptomyces* sp. 4F | ALV51359.1 | 97.03 |
| gene 6471 | 518 | pat | phosphinothricin N-acetyltransferase | *Streptomyces werraensis* | GHE99025.1 | 94.19 |
| gene 4600 | 1322 | - | 4-methylmuconolactone transporter | *Streptomyces afghaniensis* 772 | EPJ35848.1 | 87.02 |

**Table S7** Genes responsible for gentisate pathways.

| Gene ID | Size (aa) | Gene Name | Encode protein | Species of reference gene | Accession no. (NCBI) | BLAST identity (%) |
| --- | --- | --- | --- | --- | --- | --- |
| gene 1961 | 2366 | - | bifunctional salicylyl-CoA 5-hydroxylase/oxidoreductase | *Streptomyces* sp. 4F | ALV52714.1 | 97.07 |
| gene 0811 | 1613 | gabD | succinate-semialdehyde dehydrogenase | *Streptomyces* sp. UNC401CLCol | WP_028960221.1 | 99.81 |
| gene 4361 | 1388 | gabD | NADP-dependent succinic semialdehyde dehydrogenase | *Streptomyces cellulosae* | GHE69900.1 | 98.70 |
| gene 3797 | 1187 | - | fumarylacetoacetate hydrolase | *Streptomyces* sp. SID8376 | WP_205526335.1 | 99.75 |
| gene 2855 | 1286 | - | aromatic ring-hydroxylating dioxygenase subunit alpha | *Streptomyces* sp. GESEQ-13 | WP_137208924.1 | 99.53 |
| gene 4131 | 1280 | - | Phthalate 4,5-dioxygenase oxygenase subunit | *Streptomyces* sp. MBT84 | MBW8706675.1 | 70.79 |
| gene 4137 | 620 | - | gentisate 1,2-dioxygenase | *Robiginitomaculum* sp. | PHR56577.1 | 81.03 |
| gene 4928 | 1085 | frmA | alcohol dehydrogenase | *Streptomyces cellulosae* | GHE58756.1 | 97.23 |
| gene 5219 | 689 | - | maleylpyruvate isomerase | *Streptomyces* sp. XHT-2 | WP_161107348.1 | 99.13 |

**Table S8** Genes responsible for pathways for anthranilate.

| Gene ID | Size (aa) | Gene Name | Encode protein | Species of reference gene | Accession no. (NCBI) | BLAST identity (%) |
| --- | --- | --- | --- | --- | --- | --- |
| gene 1963 | 1610 | abmG | 2-aminobenzoate-CoA ligase | *Streptomyces* sp. 4F | ALV52716.1 | 94.03 |
| gene 1964 | 1128 | - | acyl-CoA dehydrogenase | *Streptomyces* sp. XHT-2 | WP_161107119.1 | 99.47 |
| gene 0037 | 638 | pabA | aminodeoxychorismate/anthranilate synthase component II | *Streptomyces* sp. GESEQ-13 | WP_210635465.1 | 99.53 |
| gene 5387 | 1880 | phzE | anthranilate synthase | *Streptomyces* sp. UNC401CLCol | WP_028960786.1 | 99.52 |
| gene 0262 | 1202 | kynU | L-kynurenine hydrolase | *Streptomyces* sp. UNC401CLCol | WP_028959521.1 | 99.25 |
| gene 0261 | 830 | TDO2 | tryptophan 2,3-dioxygenase | *Streptomyces* sp. GESEQ-13 | WP_210635572.1 | 99.28 |
| gene 4644 | 1697 | - | Tryptophan 2-monooxygenase | *Streptomyces* sp. di50b | SCD87347.1 | 97.35 |
| gene 0499 | 1217 | - | Aromatic-amino-acid aminotransferase 1 | *Streptomyces* sp. UNC401CLCol | WP_064743320.1 | 99.49 |
| gene 2090 | 873 | - | indole-3-glycerol phosphate synthase | *Streptomyces* sp. I4 (2020) | WP_199204736.1 | 96.82 |
| gene 5302 | 806 | trpC | indole-3-glycerol phosphate synthase | *Streptomyces* sp. I4 (2020) | WP_199207289.1 | 99.63 |
| gene 5306 | 1493 | trpE | anthranilate synthase component I | *Streptomyces* sp. XHT-2 | WP_161107330.1 | 99.8 |

**Table S9** Genes responsible for pathways for resorcinol.

| Gene ID | Size (aa) | Gene Name | Encode protein | Species of reference gene | Accession no. (NCBI) | BLAST identity (%) |
| --- | --- | --- | --- | --- | --- | --- |
| gene 0128 | 971 | - | LysR family transcriptional regulator | *Streptomyces* sp. UNC401CLCol | WP_078611393.1 | 99.69 |
| gene 2842 | 1208 | - | β-ketoadipyl CoA thiolase | *Streptomyces* sp. B9173 | OQR62711.1 | 88.25 |
| gene 1964 | 1128 | - | acyl-CoA dehydrogenase | *Streptomyces* sp. XHT-2 | WP_161107119.1 | 99.47 |
| gene 2768 | 1091 | - | aryl-alcohol dehydrogenase | *Streptomyces* sp. di50b | SCD35921.1 | 93.66 |
| gene 2849 | 962 | pdxA | Terephthalate dihydrodiol dehydrogenase | *Streptomyces* sp. McG7 | WP_215047915.1 | 98.75 |
| gene 2850 | 470 | andAd | terephthalate 1,2-dioxygenase | *Streptomyces* sp. XHT-2 | MXQ61646.1 | 100.00 |
| gene 2851 | 1283 | andAc | Terephthalate 1,2-dioxygenase | *Rhodococcus pyridinivorans* AK37 | EHK83592.1 | 83.05 |
| gene 3600 | 1502 | - | 2-polyprenyl-6-methoxyphenol hydroxylase | *Streptomyces* sp. di50b | SCD47389.1 | 85.06 |
| gene 5989 | 1268 | - | dimethylaniline monooxygenase | *Streptomyces* sp. 4F | ALV54407.1 | 96.91 |
| gene 3045 | 383 | - | extradiol dioxygenase | *Streptomyces* sp. 4F | ALV53653.1 | 93.70 |
| gene 4526 | 476 | - | putative dehydrochlorinase | *Streptomyces* sp. | AUR34009.1 | 81.65 |
| gene 5542 | 1025 | - | Trans-1,2-dihydrobenzene-1,2-diol dehydrogenase | *Streptomyces afghaniensis* 772 | EPJ42281.1 | 82.27 |
| gene 6472 | 785 | - | extradiol ring-cleavage dioxygenase | *Streptomyces griseorubens* | KEG41654.1 | 98.85 |

**Table S10** Genes responsible for catabolic pathways for homogentisic.

| Gene ID | Size (aa) | Gene Name | Encode protein | Species of reference gene | Accession no. (NCBI) | BLAST identity (%) |
| --- | --- | --- | --- | --- | --- | --- |
| gene 4937 | 1361 | hmgA | homogentisate 1,2-dioxygenase | *Streptomyces* sp. XHT-2 | WP_161108369.1 | 99.78 |
| gene 1961 | 2366 | - | bifunctional salicylyl-CoA 5-hydroxylase/oxidoreductase | *Streptomyces* sp. 4F | ALV52714.1 | 97.07 |
| gene 0499 | 1217 | - | Aromatic-amino-acid aminotransferase 1 | *Streptomyces* sp. UNC401CLCol | WP_064743320.1 | 99.49 |
| gene 6956 | 1079 | hisC | Putative phenylalanine aminotransferase | *Streptomyces werraensis* | GHF12148.1 | 96.93 |
| gene 0811 | 1613 | gabD | succinate-semialdehyde dehydrogenase | *Streptomyces* sp. UNC401CLCol | WP_028960221.1 | 99.81 |
| gene 4361 | 1388 | gabD | NADP-dependent succinic semialdehyde dehydrogenase | *Streptomyces cellulosae* | GHE69900.1 | 98.70 |
| gene 0636 | 1214 | fahA | Fumarylacetoacetate hydrolase | *Streptomyces* sp. SID4956 | WP_161232839.1 | 99.01 |
| gene 3797 | 1187 | - | fumarylacetoacetate hydrolase | *Streptomyces* sp. SID8376 | WP_205526335.1 | 99.75 |
| gene 2443 | 305 | phhB | 4a-hydroxytetrahydrobiopterin dehydratase | *Streptomyces griseorubens* | WP_033273716.1 | 98.02 |
| gene 4811 | 2576 | pheT | phenylalanine--tRNA ligase subunit beta | *Streptomyces* sp. GESEQ-13 | WP_210636334.1 | 99.77 |
| gene 4812 | 1121 | pheS | phenylalanine--tRNA ligase subunit alpha | *Streptomyces cellulosae* | GHE73990.1 | 100 |
| gene 3632 | 1814 | hppD | 4-hydroxyphenylpyruvate dioxygenase | *Streptomyces cellulosae* | GHE74791.1 | 97.35 |
| gene 3633 | 876 | aroE | shikimate dehydrogenase | *Streptomyces* sp. FxanaD5 | WP_019522987.1 | 99.66 |
| gene 6183 | 1145 | hppD | 4-hydroxyphenylpyruvate dioxygenase | *Actinospica acidiphila* | WP_163089359.1 | 98.69 |
| gene 4928 | 1085 | frmA | alcohol dehydrogenase | *Streptomyces cellulosae* | GHE58756.1 | 97.23 |
| gene 5219 | 689 | - | maleylpyruvate isomerase | *Streptomyces* sp. XHT-2 | WP_161107348.1 | 99.13 |

**Table S11** Genes responsible for pathways for phenylacetate-CoA.

| Gene ID | Size (aa) | Gene Name | Encode protein | Species of reference gene | Accession no. (NCBI) | BLAST identity (%) |
| --- | --- | --- | --- | --- | --- | --- |
| gene 0050 | 1061 | paaE | phenylacetic acid degradation protein | *Streptomyces cellulosae* | GHE31167.1 | 99.15 |
| gene 0051 | 539 | paaD | phenylacetate-CoA oxygenase subunit | *Streptomyces cellulosae* | GHE31162.1 | 97.77 |
| gene 0052 | 737 | paaC | phenylacetate-CoA oxygenase subunit | *Streptomyces* sp. GESEQ-13 | WP_210635474.1 | 98.78 |
| gene 0053 | 287 | paaB | phenylacetate-CoA oxygenase subunit PaaB | *Streptomyces griseoruber* | WP_055636201.1 | 98.95 |
| gene 0054 | 1016 | paaA | phenylacetate-CoA oxygenase subunit PaaA | *Streptomyces* sp. SID4956 | WP_161232185.1 | 99.41 |
| gene 0058 | 1691 | PaaN | phenylacetic acid degradation protein | *Streptomyces* sp. Akac8 | WP_136238706.1 | 99.82 |
| gene 0059 | 1514 | paaH | 3-hydroxyacyl-CoA dehydrogenase | *Streptomyces* sp. SID4956 | WP_161233084.1 | 100.00 |
| gene 1159 | 803 | paaG | 1,2-epoxyphenylacetyl-CoA isomerase | *Streptomyces cellulosae* | GHE51256.1 | 98.13 |
| gene 1381 | 848 | paaH | 3-hydroxybutyryl-CoA dehydrogenase | *Streptomyces* sp. F-7 | WP_093768152.1 | 99.65 |
| gene 2386 | 1805 | paaH | 3-hydroxybutyryl-CoA dehydrogenase | *Streptomyces* sp. SMS_SU21 | WP_102640749.1 | 97.17 |
| gene 2736 | 764 | paaF | 3-hydroxypropionyl-CoA dehydratase | *Streptomyces* sp. GESEQ-13 | WP_210638121.1 | 99.61 |
| gene 4099 | 1523 | paaK | phenylacetate-CoA ligase | *Streptomyces* sp. GESEQ-13 | WP_210637941.1 | 99.77 |
| gene 4804 | 860 | paaH | 3-hydroxybutyryl-CoA dehydrogenase | *Streptomyces* sp. I4 (2020) | WP_199207038.1 | 99.30 |
| gene 6544 | 734 | paaF | enoyl-CoA hydratase | *Streptomyces cellulosae* | GHE27114.1 | 98.77 |
| gene 4828 | 1376 | Pad | Phenylacetaldehyde dehydrogenase | *Streptomyces* sp. DI166 | SBT93309.1 | 87.53 |
| gene 1454 | 767 | - | enoyl-CoA hydratase | *Streptomyces* sp. Akac8 | WP_136239245.1 | 99.61 |
| gene 1960 | 827 | - | enoyl-CoA hydratase | *Streptomyces griseorubens* | WP_033274100.1 | 97.82 |
| gene 1962 | 846 | PaaX | phenylacetic acid degradation operon negative regulatory protein | *Streptomyces aureorectus* | MBA8975440.1 | 90.71 |

**Table S.12** Genes responsible for catabolic pathways for 2,3-dihydroxyphenylpropionate.

| Gene ID | Size (aa) | Gene Name | Encode protein | Species of reference gene | Accession no. (NCBI) | BLAST identity (%) |
| --- | --- | --- | --- | --- | --- | --- |
| gene 3746 | 1028 | - | 3-hydroxycinnamic acid hydroxylase | *Streptomyces albogriseolus* | GHB97425.1 | 91.81 |
| gene 1753 | 1640 | mhpA | 3-(3-hydroxy-phenyl) propionate acid hydroxylase | *Streptomyces* sp. di50b | SCE12787.1 | 96.15 |
| gene 2825 | 1613 | mhpA | 3-(3-hydroxy-phenyl) propionate hydroxylase | *Streptomyces griseorubens* | GGQ79721.1 | 97.77 |
| gene 2828 | 794 | mhpD | fumarylacetoacetate hydrolase | *Streptomyces* sp. GESEQ-13 | WP_210638174.1 | 99.62 |
| gene 2830 | 935 | mhpB | 3-carboxyethylcatechol 2,3-dioxygenase | *Streptomyces* sp. GESEQ-13 | WP_210638056.1 | 99.04 |
| gene 2826 | 773 | hpaI | 4-hydroxy-2-oxovalerate aldolase | *Streptomyces* sp. XHT-2 | MXQ61669.1 | 99.61 |
| gene 5487 | 359 | hpaF | isomerase | *Streptomyces cellulosae* | GHE29375.1 | 97.48 |
| gene 0171 | 431 | - | aldehyde dehydrogenase | *Streptomyces werraensis* | GHF03416.1 | 88.46 |
| gene 0388 | 1460 | - | aldehyde dehydrogenase | *Streptomyces* sp. GESEQ-13 | WP_210635659.1 | 99.18 |
